# Supplementary material for: DeepCLIP: predicting the effect of mutations on protein–RNA binding with deep learning
Source: Nucleic Acids Res. 2020 Jun 19;48(13):7099–118. doi: 10.1093/nar/gkaa530 (PMC7367176; doi:10.1093/nar/gkaa530)
Supplement: gkaa530_Supplemental_Files [file gkaa530_supplemental_files.zip › Supplementary_data.pdf]

SUPPLEMENTARY TABLE LEGENDS

Table S1

This table shows the model parameters used when training DeepCLIP models on the curated dataset originally used by Maticzka et al, 2014.

Table S2

This table shows performance metrics for the DeepCLIP models trained on the curated dataset originally used by Maticzka et al, 2014.

Table S3

This table shows the raw data used in Figure 1 and Figure S2. The highest AUROC score per dataset is indicated in bold.

Table S4

This table shows analysis results of DeepCLIP, GraphProt, and iDeepS predictions on eCLIP datasets from ENCODE and POSTAR2. The highest AUROC score for each dataset is indicated in bold. This table contains the raw data for Figure S5a-b.

Table S5

This table shows analysis results of DeepCLIP models trained on RNAcompete data on the corresponding curated datasets from Maticzka et al, 2014, along with previous results of RNAcontext, RCK, DeepBind, DLPRB-CNN and DLPRB-RNN. The highest AUROC score for each dataset is indicated in bold. This table contains the raw data for Figure S5c.

Table S6

This table shows DeepCLIP analysis results of exonic point mutations taken from the dataset previously published by Raponi et al, 2011. During preparation of this manuscript we uncovered some errors in the original dataset, which we corrected. This table contains the raw data for Figure 2c-d.

Table S7

This table shows DeepCLIP, GraphProt, and iDeepS analysis results of the exonic variants introduced in ACADM exon 5. This table contains the raw data for Figure 3b-c and Figure S36c-d.

Table S8

This table contains information about the oligos used for SPRi and the model estimates produced by the software applications CLAMP and scrubber, as well as the DeepCLIP prediction scores. This table contains the raw data for Figure 5c-d and Figure S44.

Table S9

This table contains DeepCLIP predictions of the two SSOs used to correct splicing of *ACADM* exon 6.

PREVIOUSLY CURATED DATASET

The curated CLIP-seq dataset used for training models and measuring the Area Under Receiver Operator Curve (AUROC) are identical to the CLIP-seq datasets used in the GraphProt paper (Maticzka et al. 2014), with the difference that all extra padding was removed, retaining only the peak area for DeepCLIP training. The complete datasets containing foreground and background sets for training and validation were downloaded from http://www.bioinf.uni-freiburg.de/Software/GraphProt. According to (Maticzka et al. 2014), the individual CLIP-seq datasets are obtained from the doRiNA database (http://dorina.mdc-berlin.de). Sequences in datasets are based on hg19.

Datasets from the doRiNA database:

- Ago2 HITS-CLIP (Kishore et al., 2011)
- ELAVL1 PAR-CLIP(A) & HITS-CLIP (Kishore et al., 2011)
- ELAVL1 PAR-CLIP(B) (Lebedeva et al., 2011)
- ELAVL1 PAR-CLIP(C) (Mukherjee et al., 2011)
- HNRNPC iCLIP (Konig et al., 2010)
- MOV10 PAR-CLIP (Sievers et al., 2012)
- SFRS1 CLIP-seq (Sanford et al., 2009)
- TDP-43 iCLIP (Tollervey et al., 2011)
- TIA1 & TIAL1 iCLIP (Wang et al., 2010)
- EWSR1, FUS & TAF15PAR-CLIP (Hoell et al., 2011)
- Ago1-4, IGF2BP1-3, PUM2 & QKI PAR-CLIP (Hafner et al., 2010)
- ALKBH5, C17ORF85, C22ORF28, CAPRIN1, ZC3H7B PAR-CLIP (Baltz et al., 2012)

Dataset not from the doRiNA database:

- PTB HITS-CLIP (Xue et al., 2009), (GSE19323)

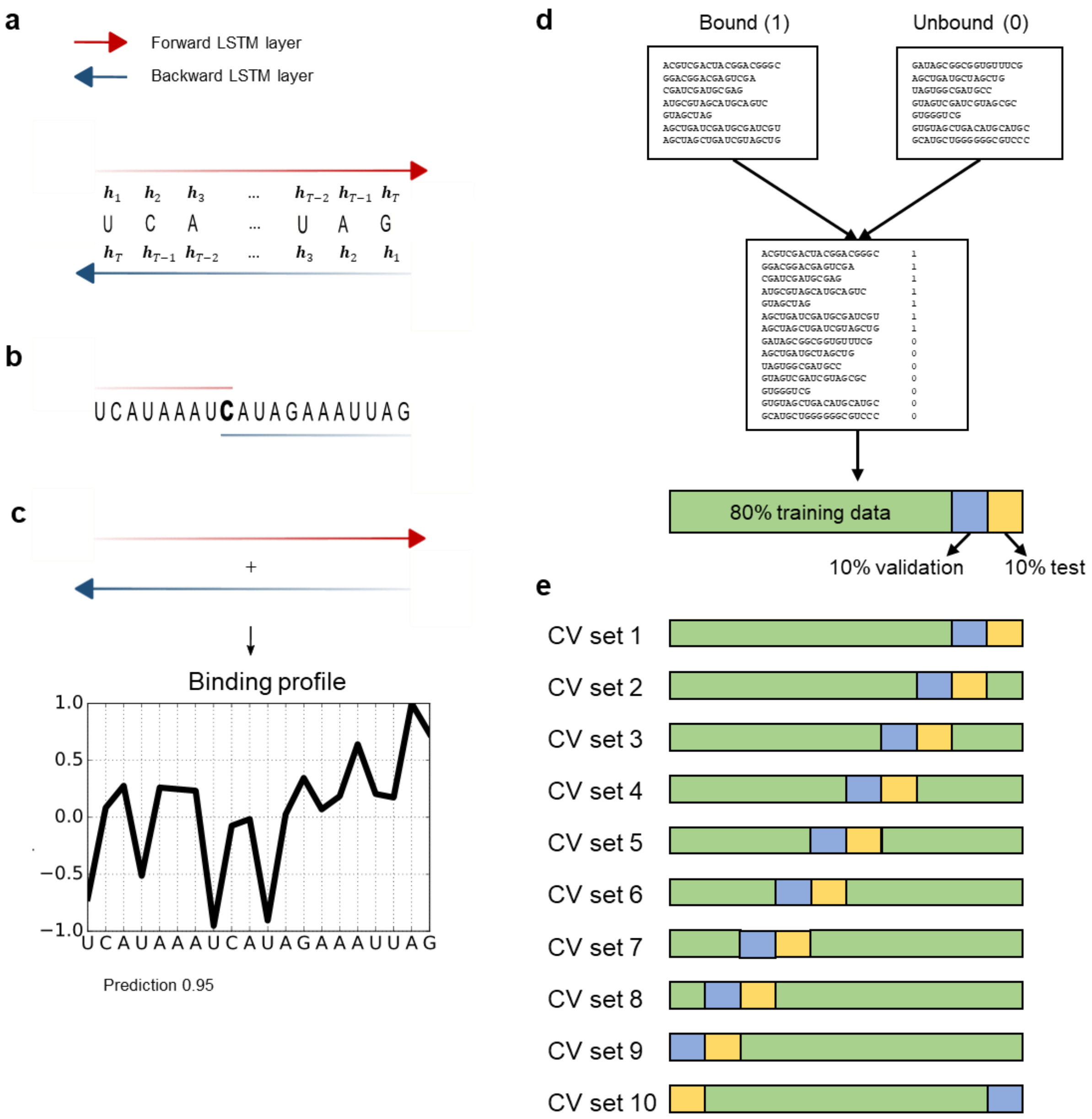

Figure S1 | Conversion of the BLSTM output to binding profile and DeepCLIP training. (a) The output of the forward LSTM layer is shown as a red arrow with a color intensity that increases gradually with the time-step number. The higher the color intensity and the higher the time-step number, the more contextual information has been available to the LSTM layer. The same is shown for the backward LSTM layer (blue arrow). The hidden state produced at the last time-step, , could contain information about the entire sequence. The outputs from the forward and backward LSTM layers are concatenated so hidden-states that are based on the base are combined. This figure is equal to from the BLSTM layer. Here, the time-step order is equal to the time-step order of the forward LSTM layer. (b) The contextual information that may present in BLSTM hidden-state that represents the highlighted cytosine (C in bold) is shown. The cytosine contains information about all the bases that surrounds it. (c) The binding profile is found via a summation of the vector elements in the BLSTM hidden-states. This results in a new vector that has a length equal to the input sequence. Every element of the vector indicates a class; positive value = class 1 (protein) and negative value = class 0 (genomic background). Every sequence has been divided by the vector element that has the highest absolute value. This results in a sequence that ranges from -1 to 1 and where it is easy to locate areas that are import for protein binding and areas that look like random genomic background. At the bottom, the prediction of the sequences in shown. (d) DeepCLIP is trained on input sequences belonging to either the bound class (assigned a score of 1) or the unbound (background) class (assigned a score of 0). The combined dataset is then divided into a training set, a validation set, and a test set. With default options they constitute 80%, 10%, and 10% of the dataset respectively.(e) When running in 10-fold cross-validation (CV) mode, the training set is divided into 10 different segmentations with a non-overlapping distribution of sequences in the validation and test set. These 10 different segmentations are then used to train 10 different models.

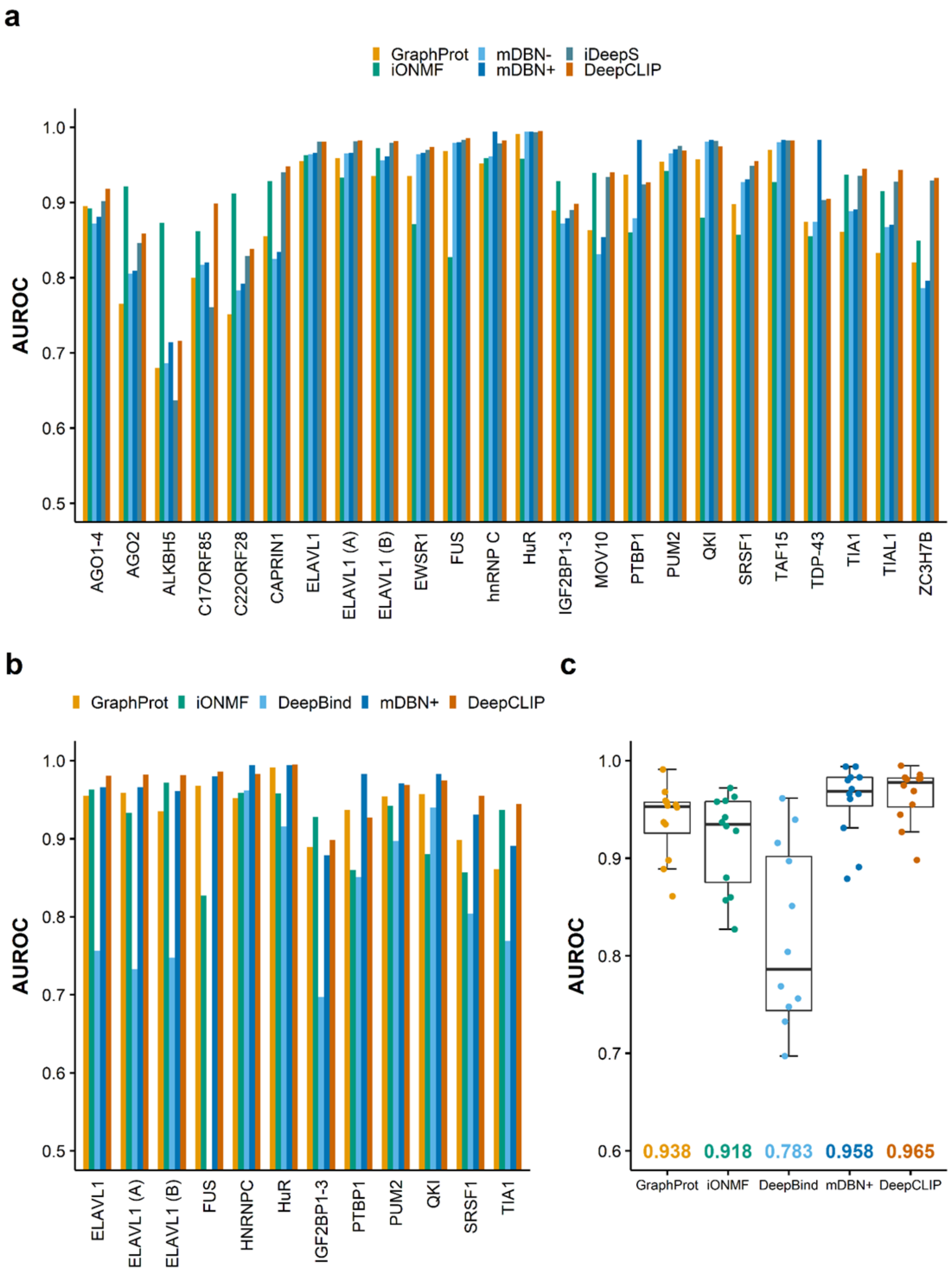

Figure S2 | Comparison of DeepCLIP AUROC performance on a benchmark dataset with existing tools. (a) Peak-sequences from the same input sequences used in Maticzka et al., 2014 were used to train DeepCLIP and iDeepS models using 10-fold cross-validation. Area under receiver operating characteristics (AUROC) for each protein were calculated using the combined predictions of all 10 models. AUROC for other methods were based on the reported scores from these studies. mDBN- indicates Deepnet with secondary structures, mDBN+ indicates Deepnet with secondary and tertiary structures. (b) AUROC metrics for the 12 proteins with DeepBind available were obtained by running DeepBind on the full dataset with all available models for the protein and using the model with the highest performance. All other values are identical to those in (a). Because deepnet with tertiary structures performed better than with just secondary structures, we show only this variant in this plot. (c) Boxplot of AUROC scores from each method for the 12 datasets. DeepBind produced an AUROC score of 0.31 for FUS, this datapoint is outside the plotting areas of both (b) and (c) plots.

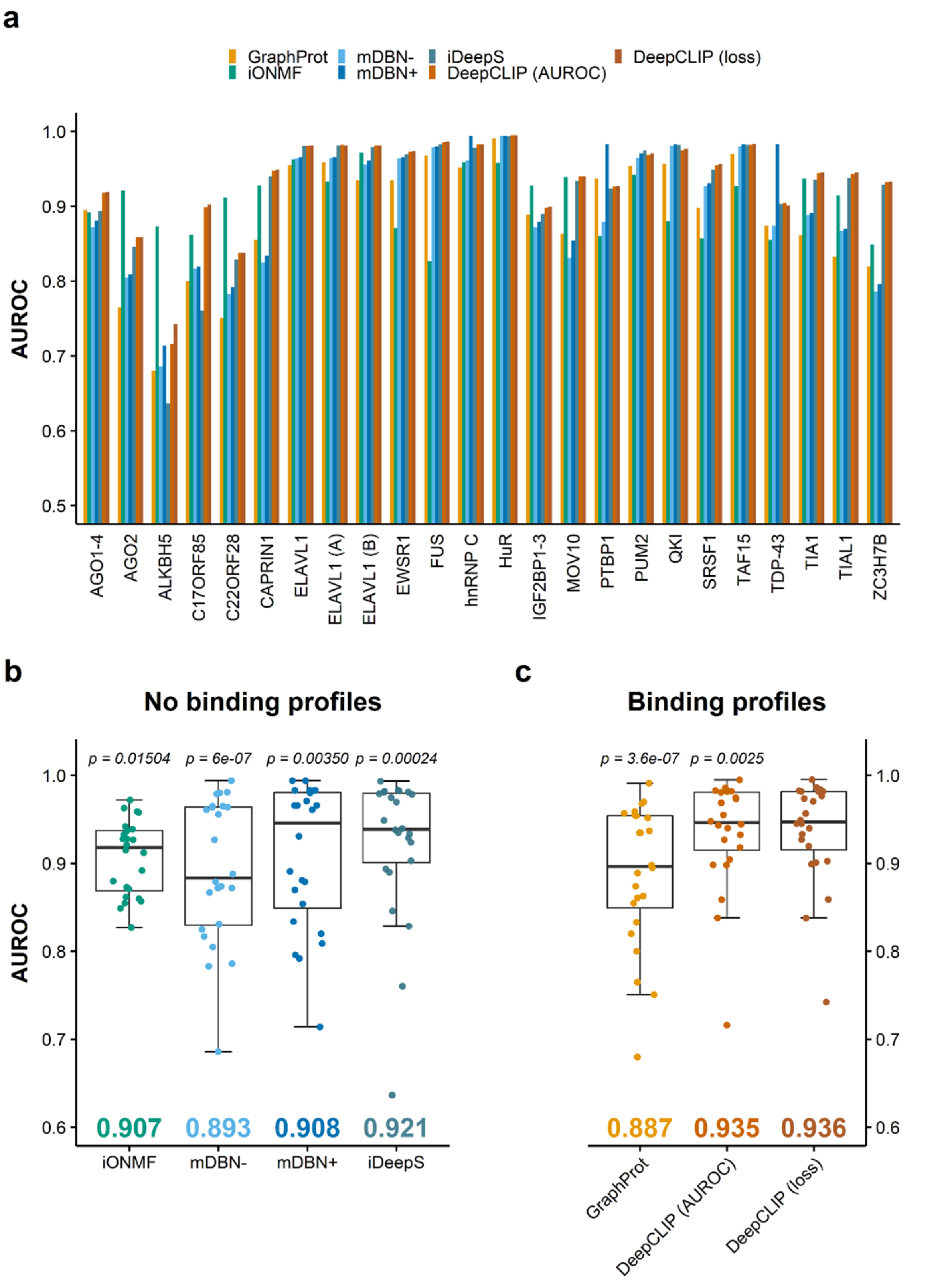

Figure S3 | Comparison of DeepCLIP with loss-selection on a benchmark dataset with existing tools. (a) Same as Figure S2a, but with addition of the DeepCLIP loss-based model performance. (b-c) Same as Figure 2C, but with addition of DeepCLIP loss-based model performance, and p-values indicate significance between the method and DeepCLIP with loss-based model selection.

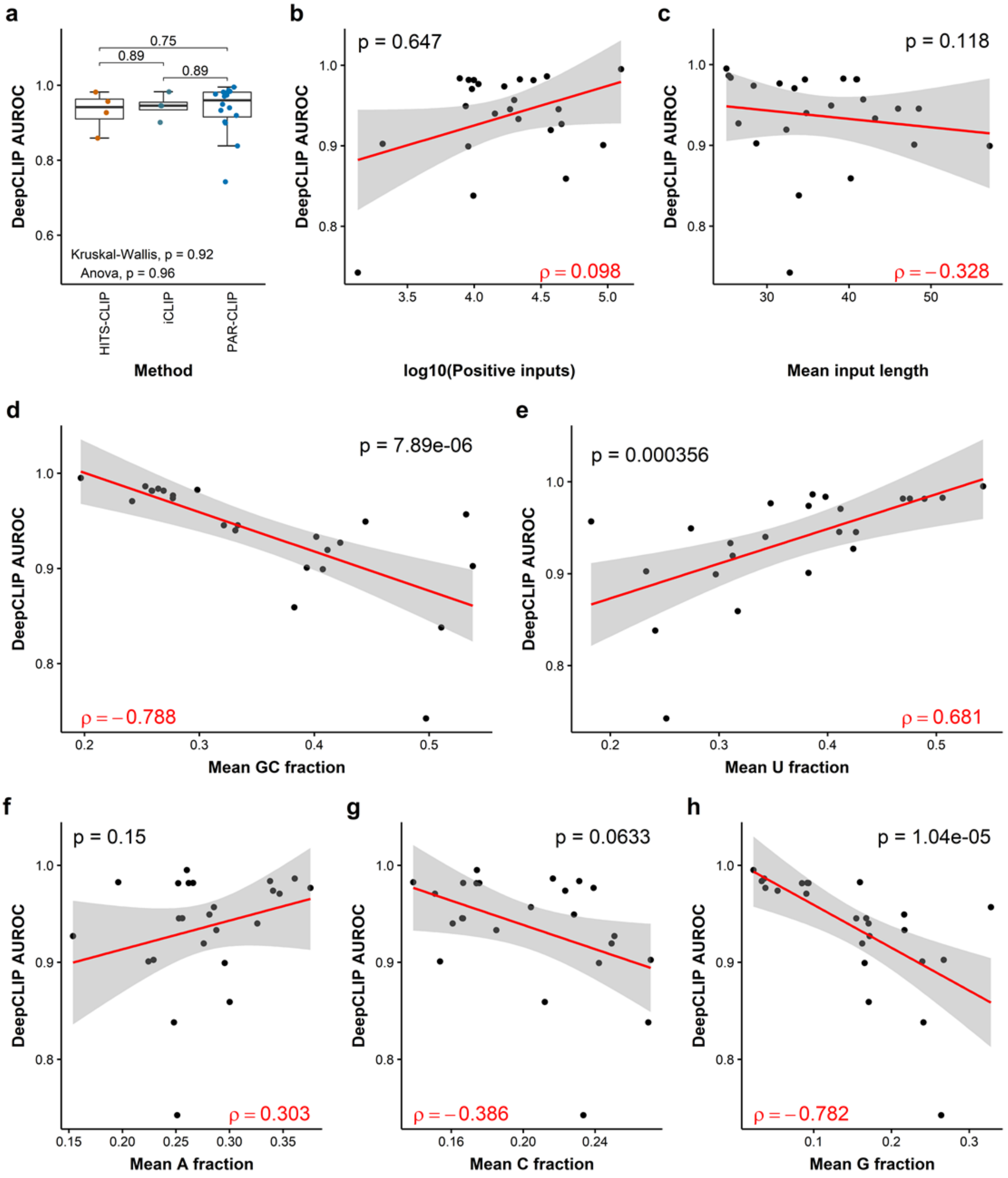

Figure S4 | DeepCLIP AUROC performance by CLIP method and dataset size. (a) AUROC metrics for DeepCLIP grouped into CLIP methods. Significance of pairwise differences was calculated using Wilcoxon rank sum tests. (b) Correlation plot of DeepCLIP AUROC measures and log10 of the number of input binding sites. Correlation was estimated using Spearman's rank correlation rho. (c) Same as (b), but correlation between mean input length and AUROC. (d) Correlation between GC-percentage and AUROC. (e) Correlation between uracil-percentage and AUROC. (f-h), same as (e), but with A-percentage, C.-percentage, and G-percentage respectively.

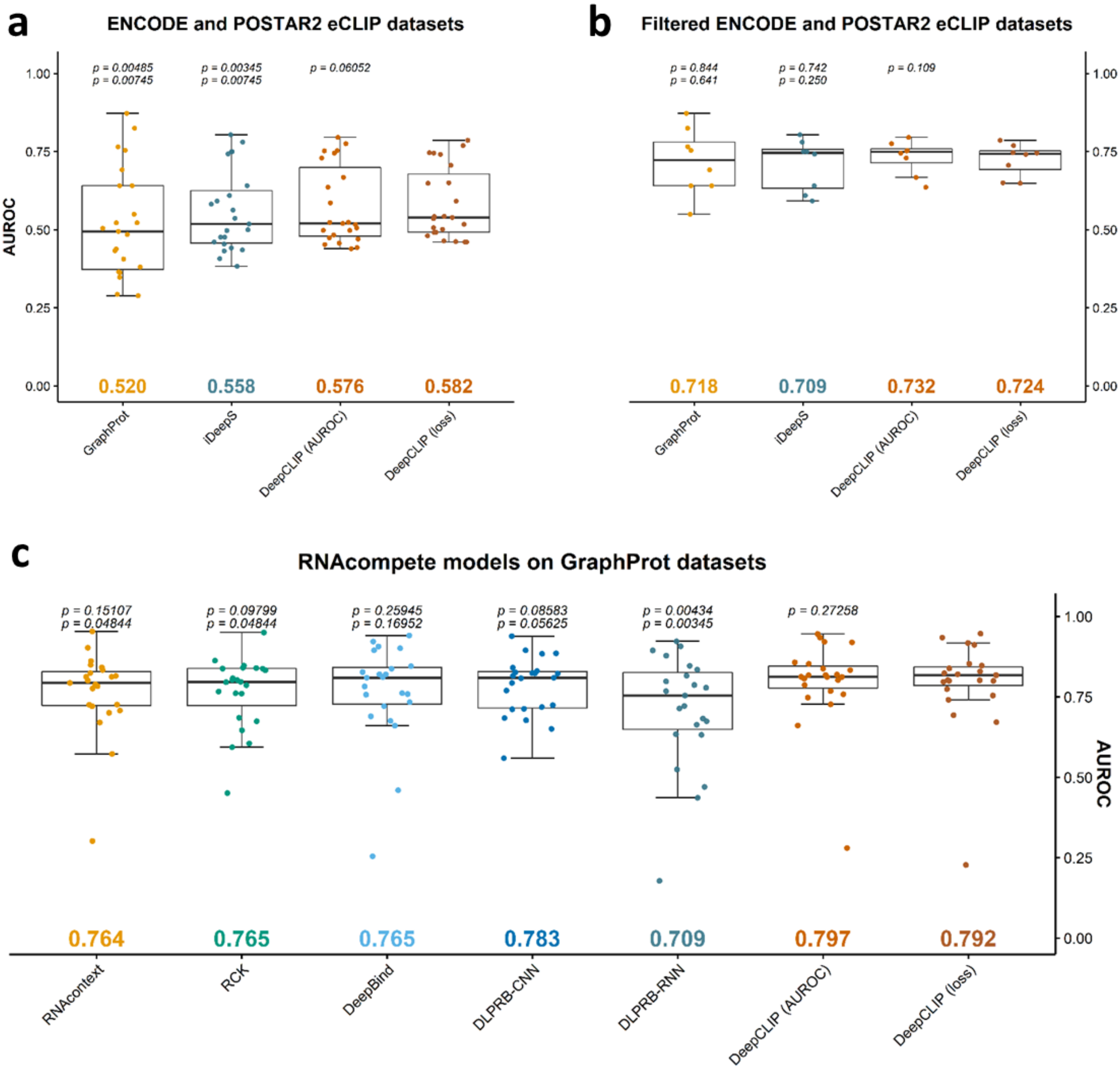

Figure S5 | Benchmark of models on independent datasets. (a) Model performance on eCLIP datasets from POSTAR2 and ENCODE measured by AUROC of GraphProt, iDeepS, DeepCLIP with AUROC based best model selection, and DeepCLIP with loss based best-model selection. The top row p-values are comparison against DeepCLIP (loss), and the bottom row against DeepCLIP (AUROC). The mean of each method is indicated at the bottom of the plot. (b) Same as (a), but only those dataset where at least one model showed AUROC > 0.6. (c) DeepCLIP models trained on the top and bottom 1000 sequences from RNAcompete experiments previously used to compare models on the GraphProt datasets (DLPRB paper). Numbers were taken from the previous study and the DeepCLIP models' performance were measured on the GraphProt datasets. At the bottom the mean AUROC performance of each method is indicated. The top row p-values are comparison (paired Wilcoxon) against DeepCLIP with loss-based model selection, and the bottom row p-values are comparison against DeepCLIP with AUROC-based model selection.

Protein (dataset)	Known binding preference	Add. binding preference source	CLIP data source	CLIP method	Top-2 CNN filters
AGO1-4	Mainly binds coding regions and 3'UTR	-	Hafner et al., 2010	PAR-CLIP	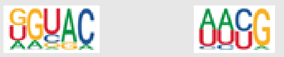
AGO2	Mainly binds coding regions and 3'UTR	-	Kishore et al., 2011	HITS-CLIP	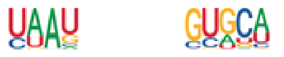
ALKBH5	Mainly binds coding regions and 3'UTR	-	Baltz et al., 2012	PAR-CLIP	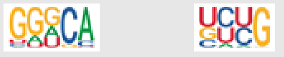
C17ORF85	Mainly binds coding regions	-	Baltz et al., 2012	PAR-CLIP	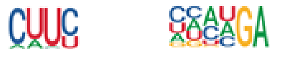
C22ORF28	Mainly binds coding regions	-	Baltz et al., 2012	PAR-CLIP	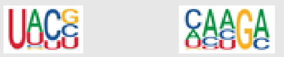
CAPRIN1	Mainly binds coding regions and 3'UTR	-	Baltz et al., 2012	PAR-CLIP	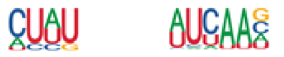
ELAVL1	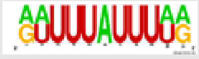	Gao et al, 1994	Kishore et al., 2011	HITS-CLIP	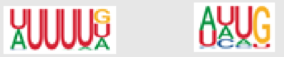
ELAVL1 (A)	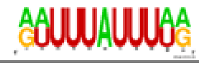	Gao et al, 1994	Kishore et al., 2011	PAR-CLIP	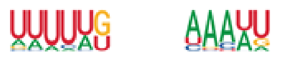
ELAVL1 (B)	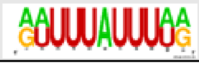	Gao et al, 1994	Lebedeva et al., 2011	PAR-CLIP	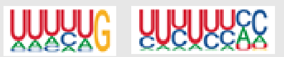
EWSR1	Binds AU-rich loop structures	-	Hoell et al., 2011	PAR-CLIP	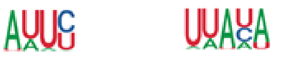
FUS	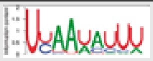	Munteanu et al, 2018	Hoell et al., 2011	PAR-CLIP	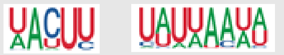
hnRNP C	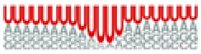	-	König et al., 2010	iCLIP	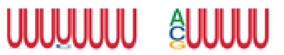
HuR	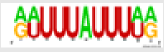	Gao et al, 1994	Mukherjee et al., 2011	PAR-CLIP	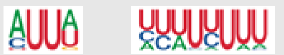
IGF2BP1-3	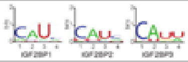	-	Hafner et al., 2010	PAR-CLIP	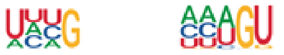
MOV10	Binds AU-rich 3' UTR regions	Gregersen et al., 2014	Sievers et al., 2012	PAR-CLIP	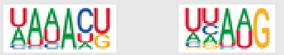
PTBP1	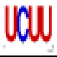	Perez et al., 1997	Xue et al., 2009	HITS-CLIP	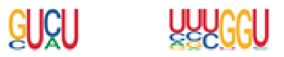
PUM2	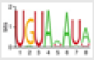	-	Hafner et al., 2010	PAR-CLIP	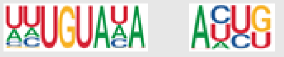
QKI	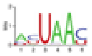	-	Hafner et al., 2010	PAR-CLIP	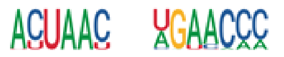
SRSF1	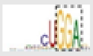	Feng et al, 2019	Sanford et al., 2009	HITS-CLIP	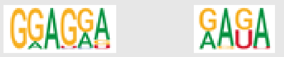
TAF15	Binds AU-rich loop structures	-	Hoell et al., 2011	PAR-CLIP	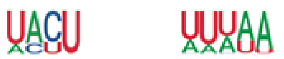
TDP-43	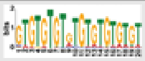	Colombrita et al, 2012	Tollervey et al., 2011	iCLIP	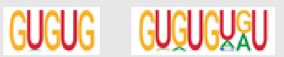
TIA1	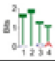 Binds U-rich regions near 5'ss	Meyer et al, 2018	Wang et al., 2010	iCLIP	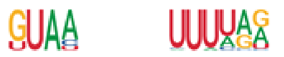
TIAL1	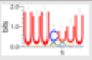 Binds U-rich regions near 5'ss	-	Wang et al., 2010	iCLIP	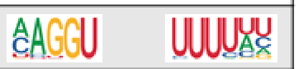
ZC3H7B	Mainly binds coding regions, 3'UTR and intronic regions	-	Baltz et al., 2012	PAR-CLIP	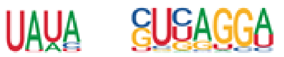

Figure S6 | Pseudo-PFMs captured by DeepCLIP. The column **Protein** contains the names of the analyzed protein/dataset, which have binding preferences as described in the column **Known binding preference**. References to literature can be seen in column 3 and 4. In the outermost right column, the top-2 by information content score **Pseudo-PFM**, motifs derived from DeepCLIPs convolutional filters are shown. H: A, C or U; N: A, C, G or U.

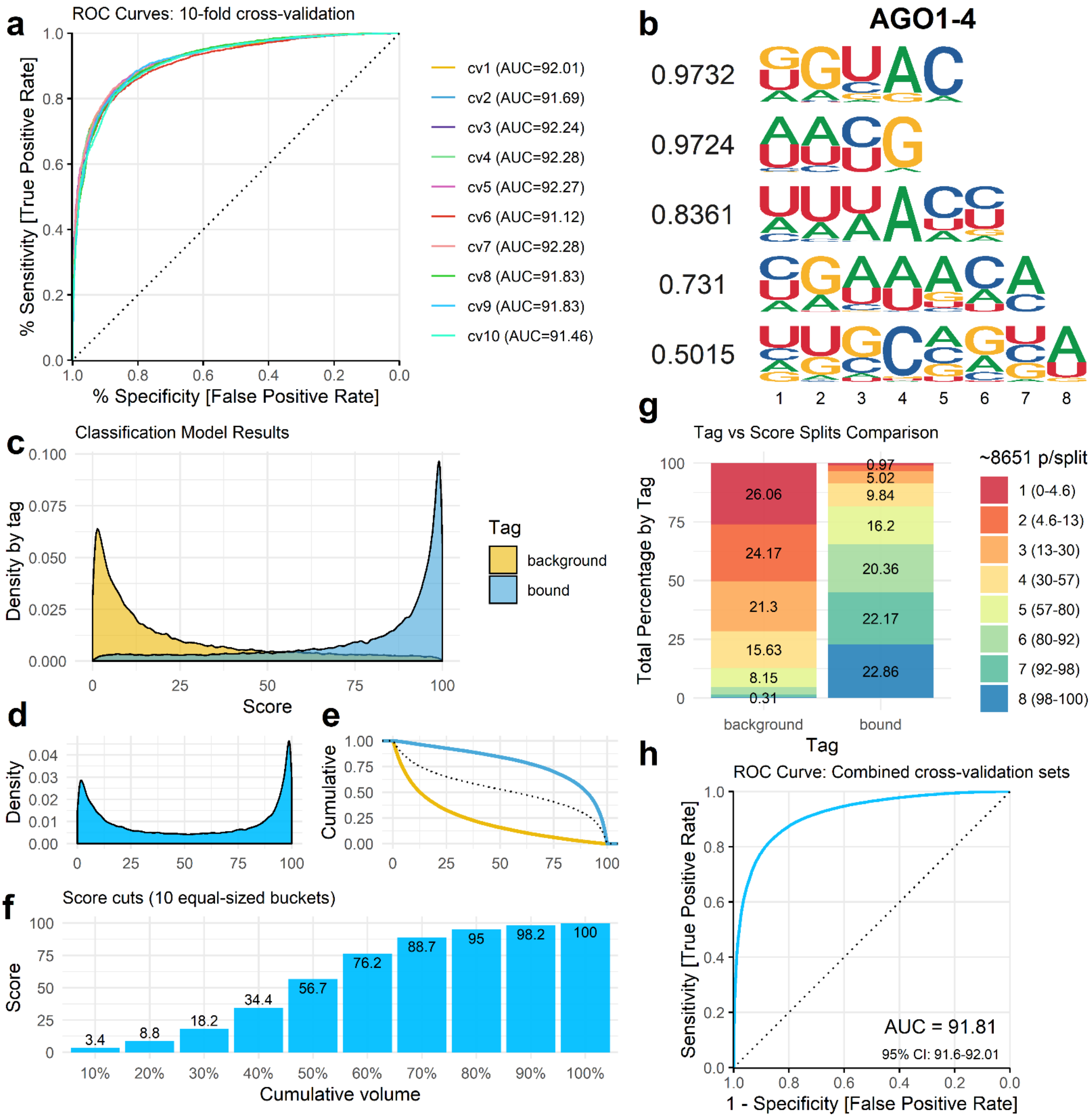

Figure S7 | DeepCLIP model characteristics for AGO1-4. (a) Area under curve analysis of DeepCLIP models trained on AGO1-4 PAR-CLIP data from Hafner et al. using 10-fold cross-validation. (b) Visualization of the CNN filters learned by the best performing model based on AUC. Score is equal to the mean information per base. (c-h) Visualizations of the combined model predictions of the 10-fold cross-validation. Scores are scaled to 0-100. (c) Density of background and bound prediction scores. (d) Combined density of prediction scores. (e) Cumulative predictive score of background and bound input sequences. (f) Barplot of cumulative scores of all input sequences. (g) Split prediction scores of background and bound input sequences. (h) Combined AUROC analysis using the pROC R package with DeLong estimation of 95% confidence interval.

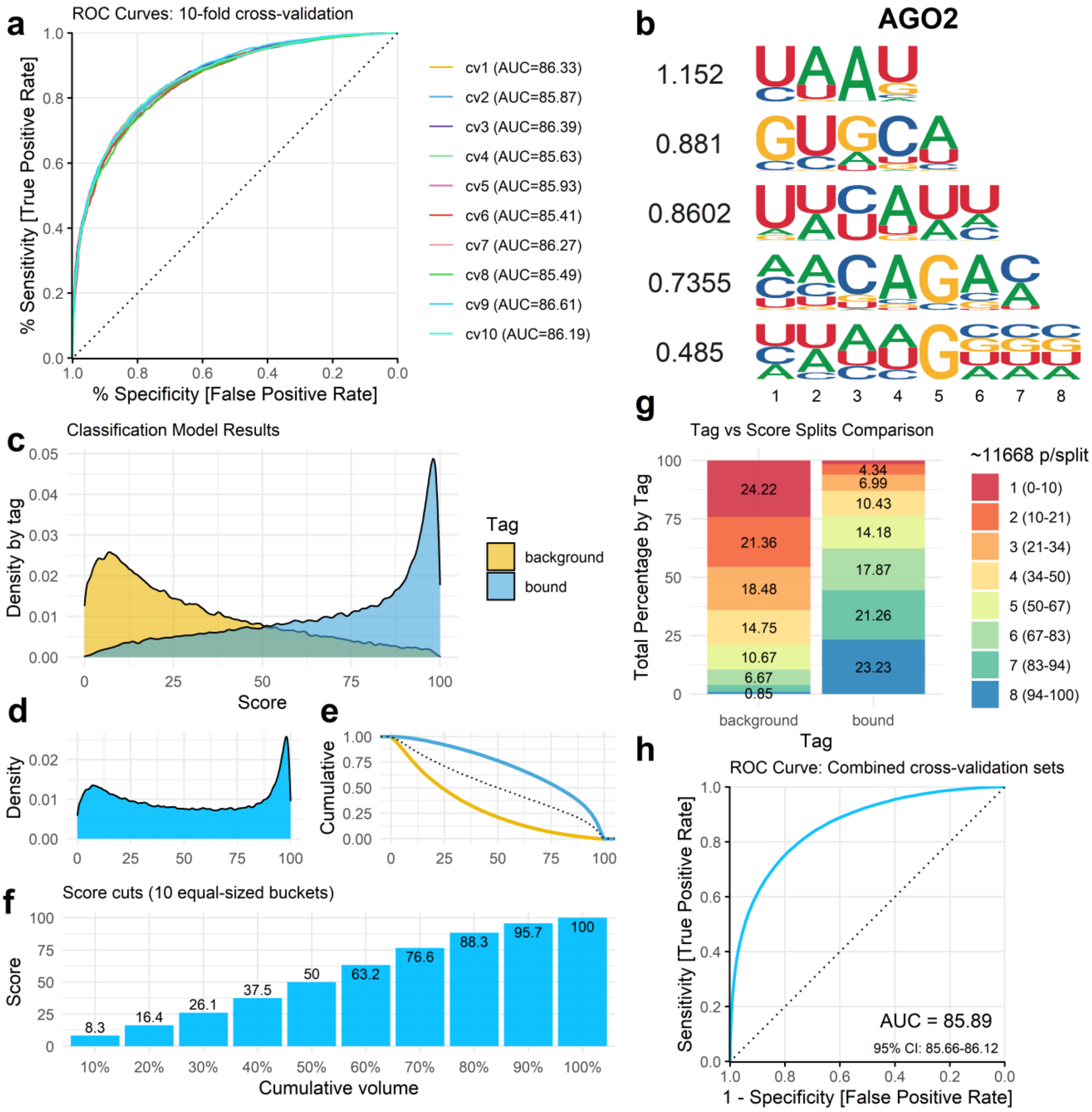

Figure S8 | DeepCLIP model characteristics for AGO2. (a) Area under curve analysis of DeepCLIP models trained on AGO2 HITS-CLIP data from Kishore et al. using 10-fold cross-validation. (b) Visualization of the CNN filters learned by the best performing model based on AUC. Score is equal to the mean information per base. (c-h) Visualizations of the combined model predictions of the 10-fold cross-validation. Scores are scaled to 0-100. (c) Density of background and bound prediction scores. (d) Combined density of prediction scores. (e) Cumulative predictive score of background and bound input sequences. (f) Barplot of cumulative scores of all input sequences. (g) Split prediction scores of background and bound input sequences. (h) Combined AUROC analysis using the pROC R package with DeLong estimation of 95% confidence interval.

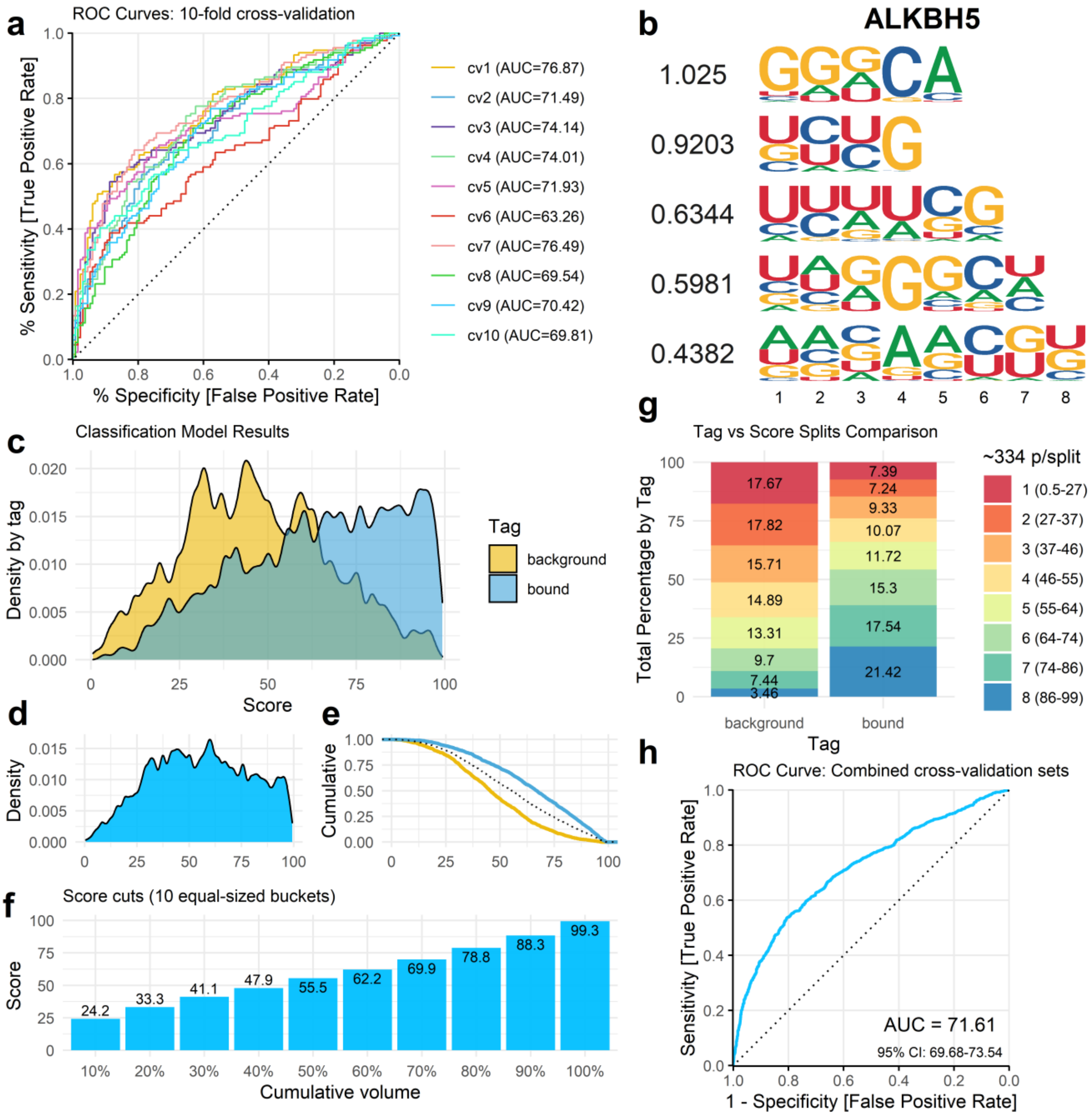

Figure S9 | DeepCLIP model characteristics for ALKBH5. (a) Area under curve analysis of DeepCLIP models trained on ALKBH5 PAR-CLIP data from Baltz et al. using 10-fold cross-validation. (b) Visualization of the CNN filters learned by the best performing model based on AUC. Score is equal to the mean information per base. (c-h) Visualizations of the combined model predictions of the 10-fold cross-validation. Scores are scaled to 0-100. (c) Density of background and bound prediction scores. (d) Combined density of prediction scores. (e) Cumulative predictive score of background and bound input sequences. (f) Barplot of cumulative scores of all input sequences. (g) Split prediction scores of background and bound input sequences. (h) Combined AUROC analysis using the pROC R package with DeLong estimation of 95% confidence interval.

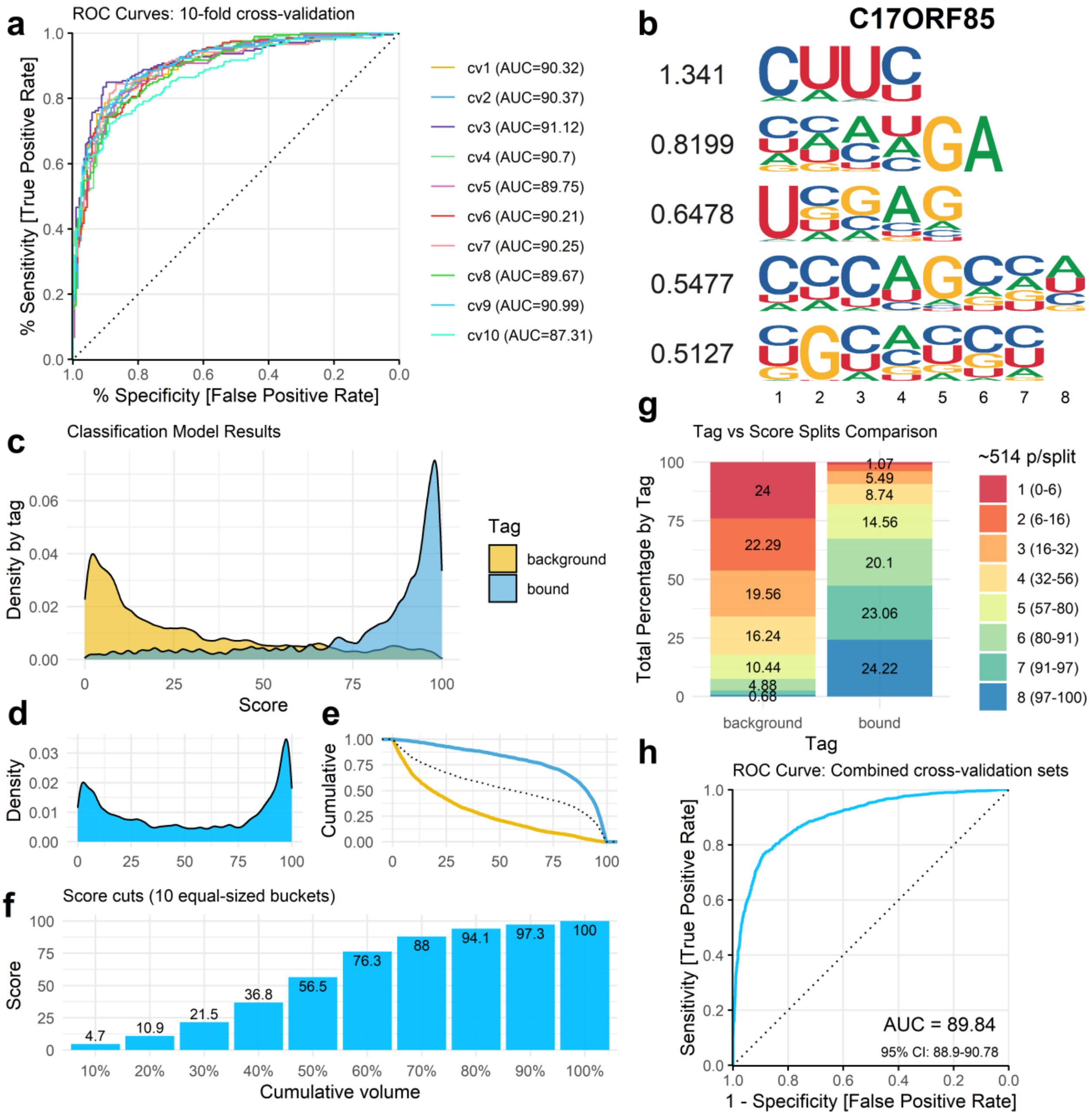

Figure S10 | DeepCLIP model characteristics for C17ORF85. (a) Area under curve analysis of DeepCLIP models trained on C17ORF85 PAR-CLIP data from Baltz et al. using 10-fold cross-validation. (b) Visualization of the CNN filters learned by the best performing model based on AUC. Score is equal to the mean information per base. (c-h) Visualizations of the combined model predictions of the 10-fold cross-validation. Scores are scaled to 0-100. (c) Density of background and bound prediction scores. (d) Combined density of prediction scores. (e) Cumulative predictive score of background and bound input sequences. (f) Barplot of cumulative scores of all input sequences. (g) Split prediction scores of background and bound input sequences. (h) Combined AUROC analysis using the pROC R package with DeLong estimation of 95% confidence interval.

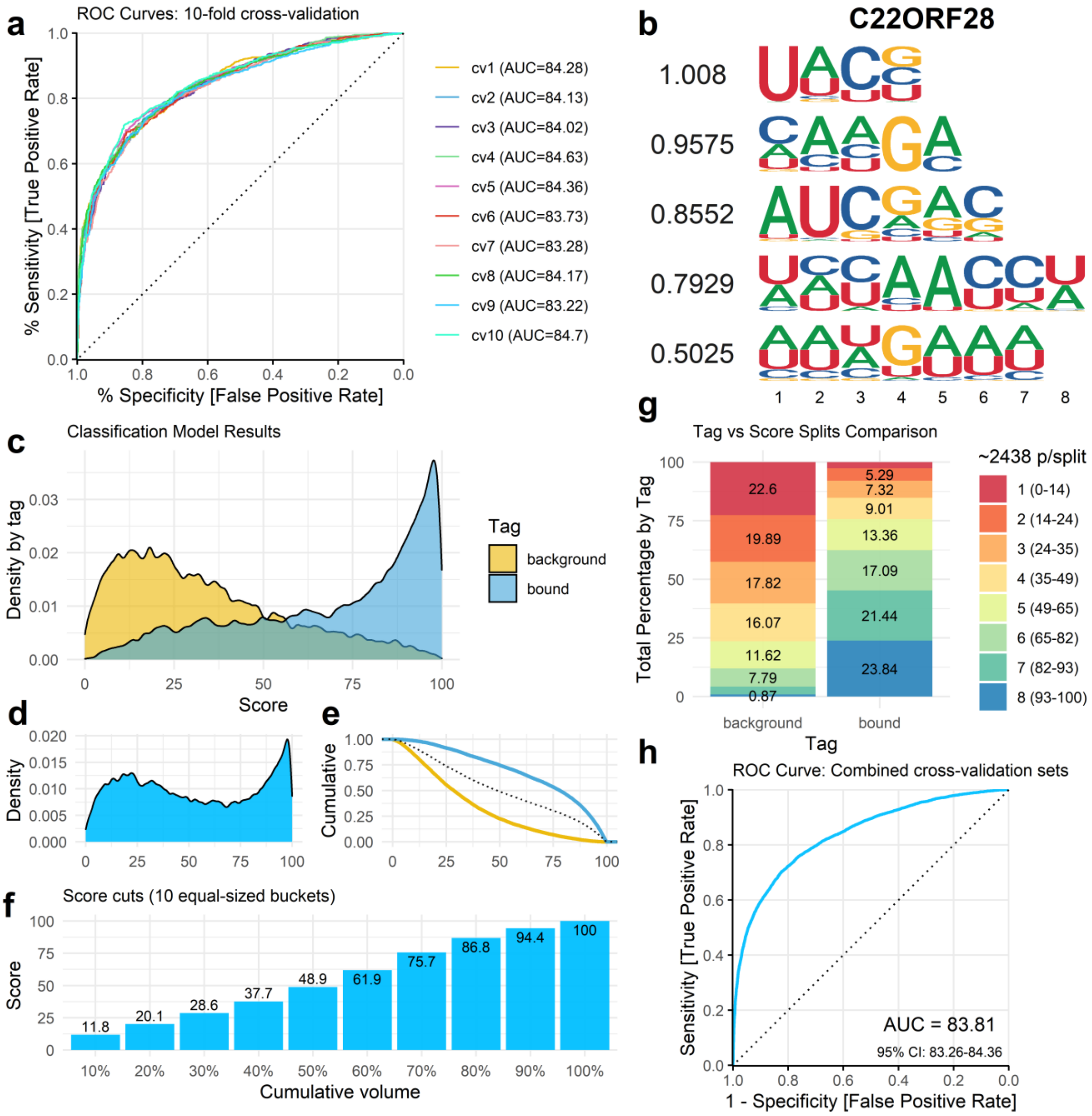

Figure S11 | DeepCLIP model characteristics for C22ORF28. (a) Area under curve analysis of DeepCLIP models trained on C22ORF28 PAR-CLIP data from Baltz et al. using 10-fold cross-validation. (b) Visualization of the CNN filters learned by the best performing model based on AUC. Score is equal to the mean information per base. (c-h) Visualizations of the combined model predictions of the 10-fold cross-validation. Scores are scaled to 0-100. (c) Density of background and bound prediction scores. (d) Combined density of prediction scores. (e) Cumulative predictive score of background and bound input sequences. (f) Barplot of cumulative scores of all input sequences. (g) Split prediction scores of background and bound input sequences. (h) Combined AUROC analysis using the pROC R package with DeLong estimation of 95% confidence interval.

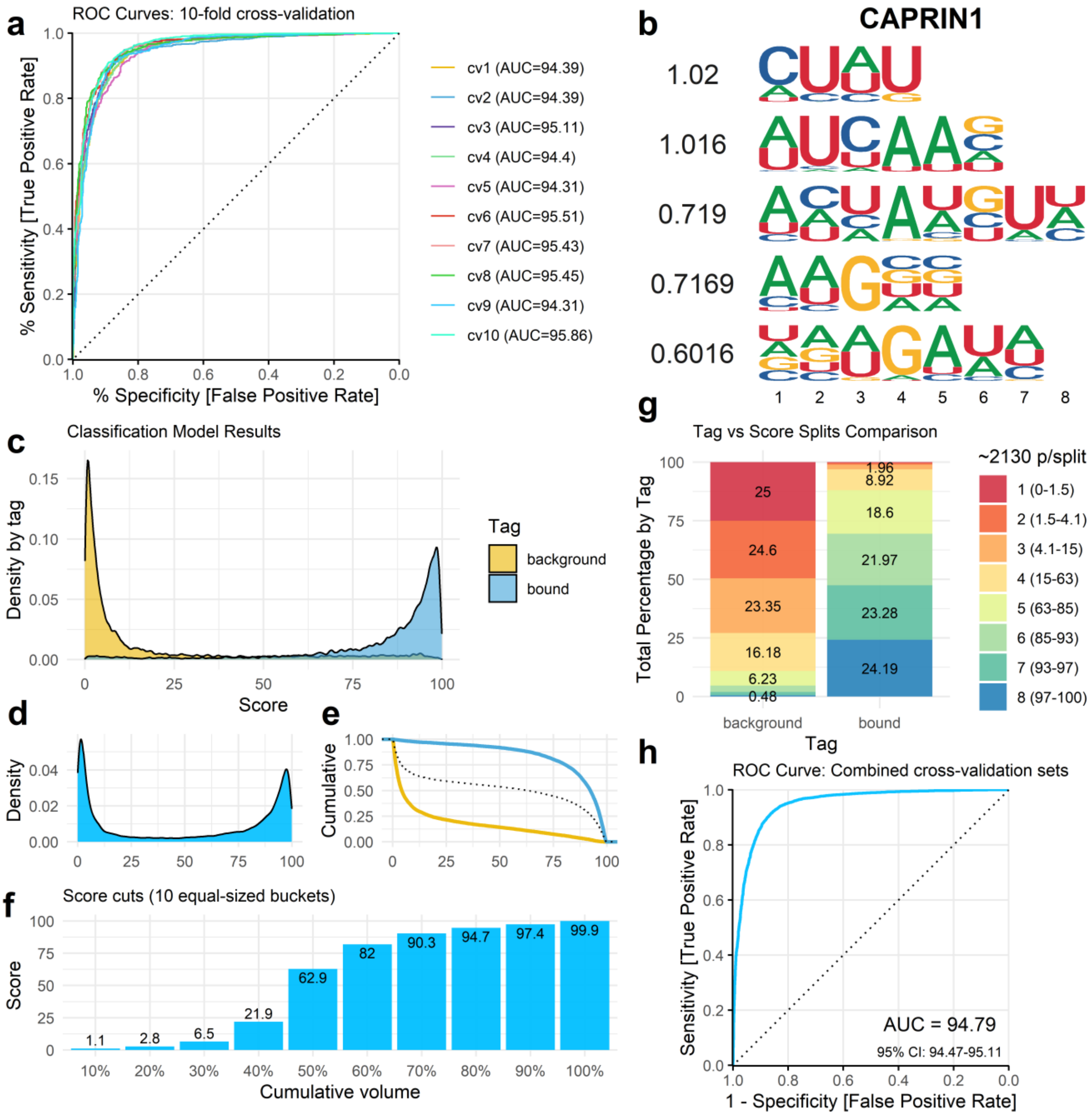

Figure S12 | DeepCLIP model characteristics for CAPRIN1. (a) Area under curve analysis of DeepCLIP models trained on CAPRIN1 PAR-CLIP data from Baltz et al. using 10-fold cross-validation. (b) Visualization of the CNN filters learned by the best performing model based on AUC. Score is equal to the mean information per base. (c-h) Visualizations of the combined model predictions of the 10-fold cross-validation. Scores are scaled to 0-100. (c) Density of background and bound prediction scores. (d) Combined density of prediction scores. (e) Cumulative predictive score of background and bound input sequences. (f) Barplot of cumulative scores of all input sequences. (g) Split prediction scores of background and bound input sequences. (h) Combined AUROC analysis using the pROC R package with DeLong estimation of 95% confidence interval.

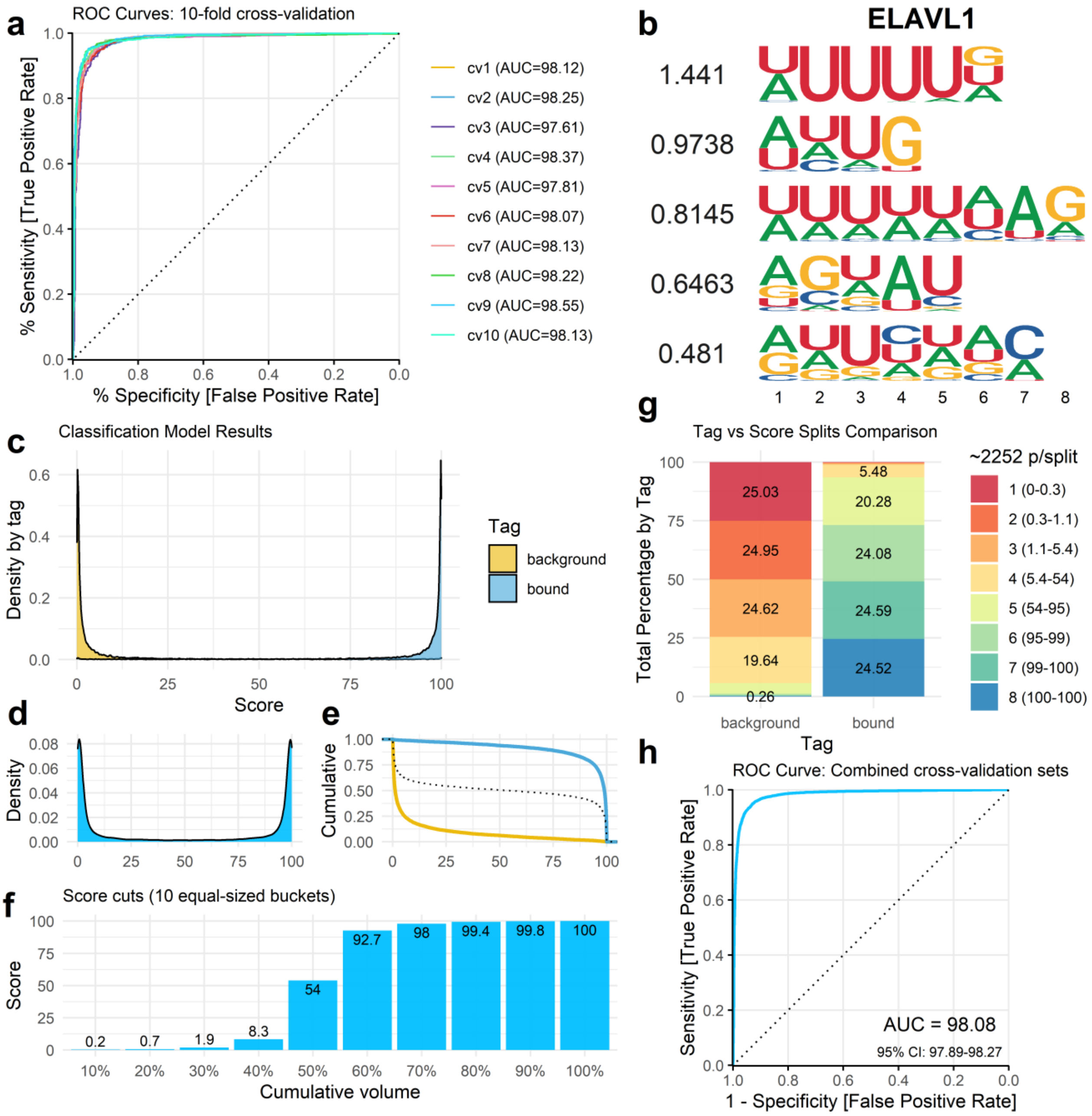

Figure S13 | DeepCLIP model characteristics for ELAVL1. (a) Area under curve analysis of DeepCLIP models trained on ELAVL1 HITS-CLIP data from Kishore et al. using 10-fold cross-validation. (b) Visualization of the CNN filters learned by the best performing model based on AUC. Score is equal to the mean information per base. (c-h) Visualizations of the combined model predictions of the 10-fold cross-validation. Scores are scaled to 0-100. (c) Density of background and bound prediction scores. (d) Combined density of prediction scores. (e) Cumulative predictive score of background and bound input sequences. (f) Barplot of cumulative scores of all input sequences. (g) Split prediction scores of background and bound input sequences. (h) Combined AUROC analysis using the pROC R package with DeLong estimation of 95% confidence interval.

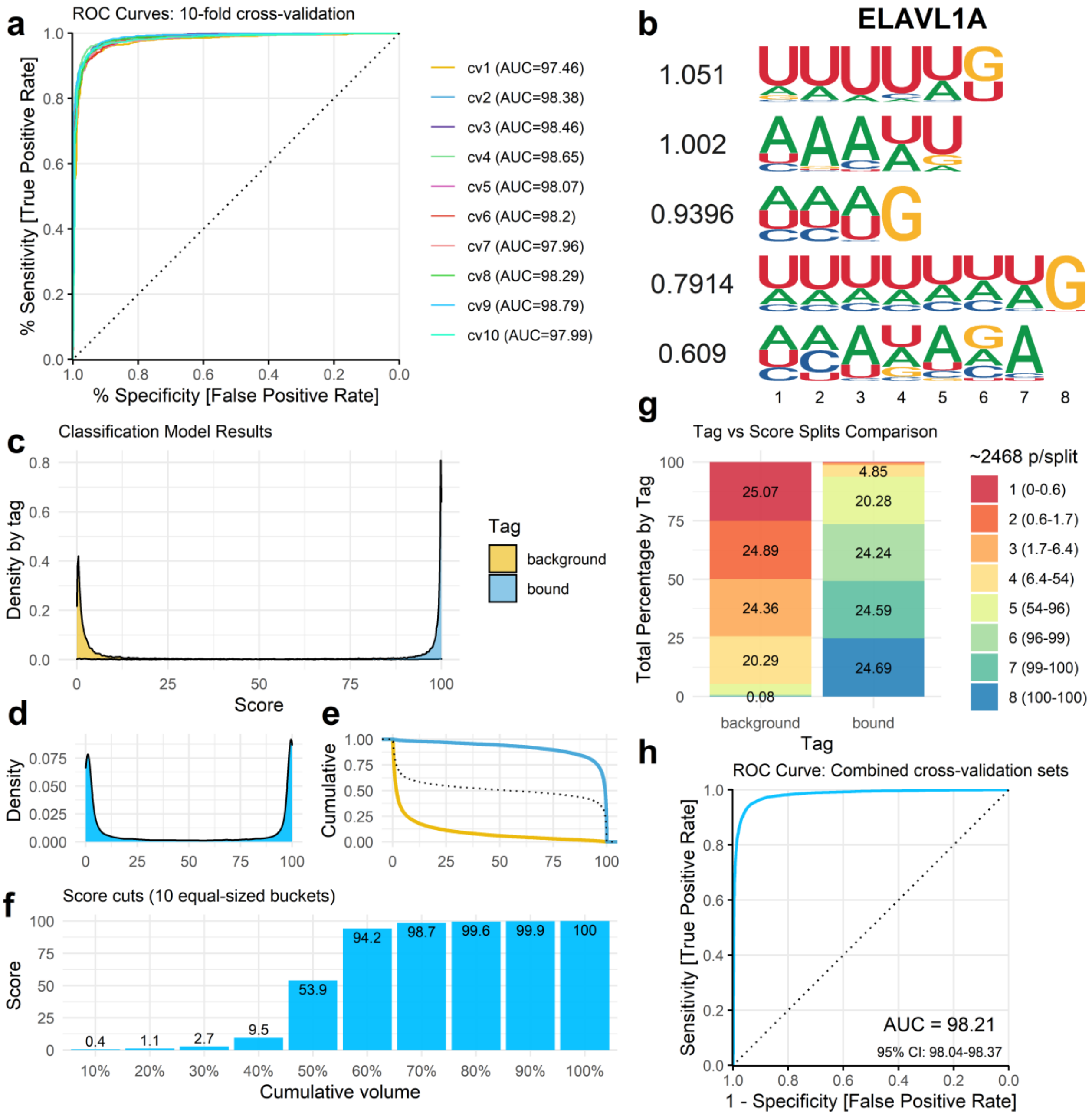

Figure S14 | DeepCLIP model characteristics for ELAVL1A. (a) Area under curve analysis of DeepCLIP models trained on ELAVL1A PAR-CLIP data from Kishore et al. using 10-fold cross-validation. (b) Visualization of the CNN filters learned by the best performing model based on AUC. Score is equal to the mean information per base. (c-h) Visualizations of the combined model predictions of the 10-fold cross-validation. Scores are scaled to 0-100. (c) Density of background and bound prediction scores. (d) Combined density of prediction scores. (e) Cumulative predictive score of background and bound input sequences. (f) Barplot of cumulative scores of all input sequences. (g) Split prediction scores of background and bound input sequences. (h) Combined AUROC analysis using the pROC R package with DeLong estimation of 95% confidence interval.

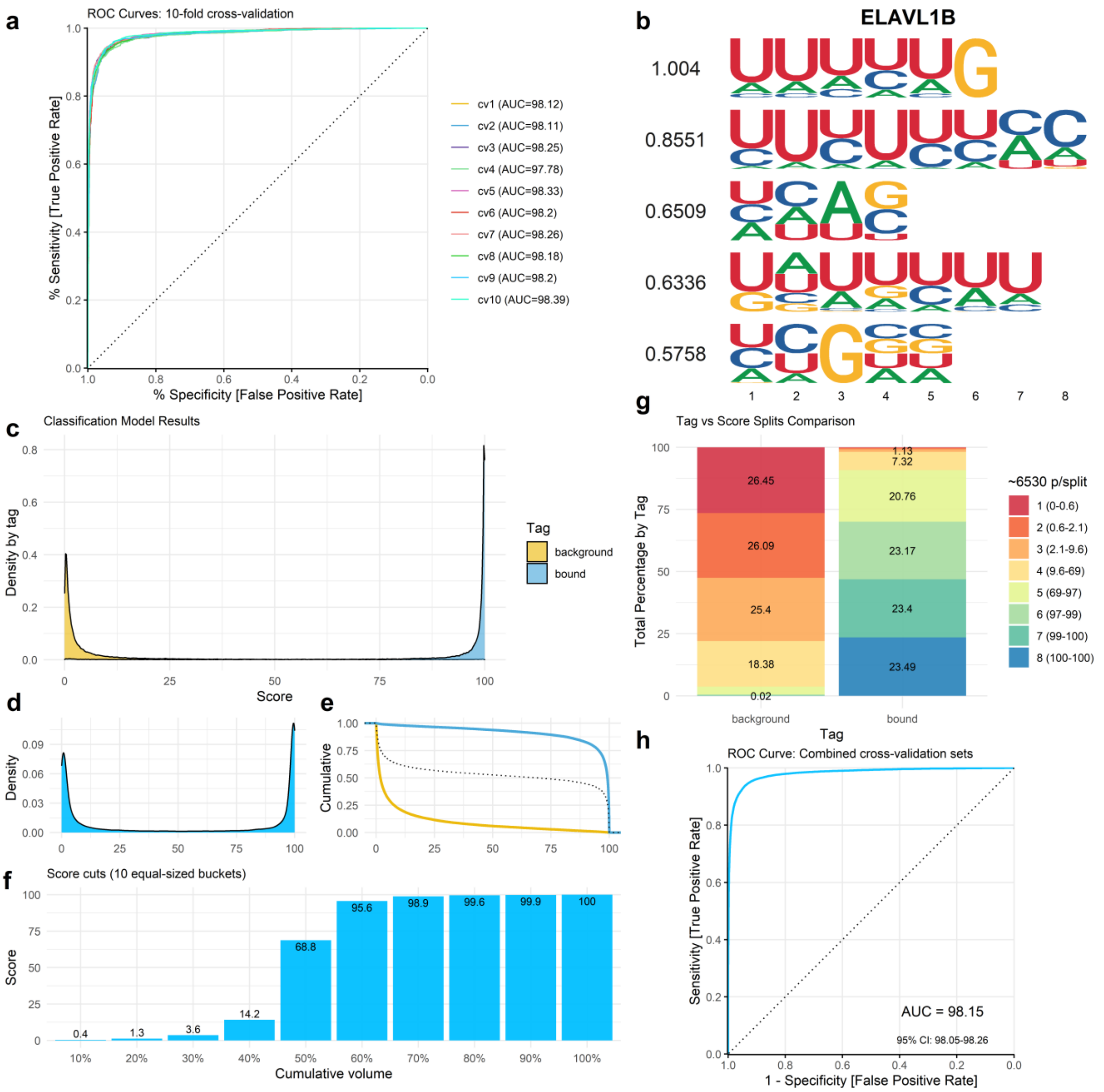

Figure S15 | DeepCLIP model characteristics for ELAVL1B. (a) Area under curve analysis of DeepCLIP models trained on ELAVL1B PAR-CLIP data from Lebedeva et al. using 10-fold cross-validation. (b) Visualization of the CNN filters learned by the best performing model based on AUC. Score is equal to the mean information per base. (c-h) Visualizations of the combined model predictions of the 10-fold cross-validation. Scores are scaled to 0-100. (c) Density of background and bound prediction scores. (d) Combined density of prediction scores. (e) Cumulative predictive score of background and bound input sequences. (f) Barplot of cumulative scores of all input sequences. (g) Split prediction scores of background and bound input sequences. (h) Combined AUROC analysis using the pROC R package with DeLong estimation of 95% confidence interval.

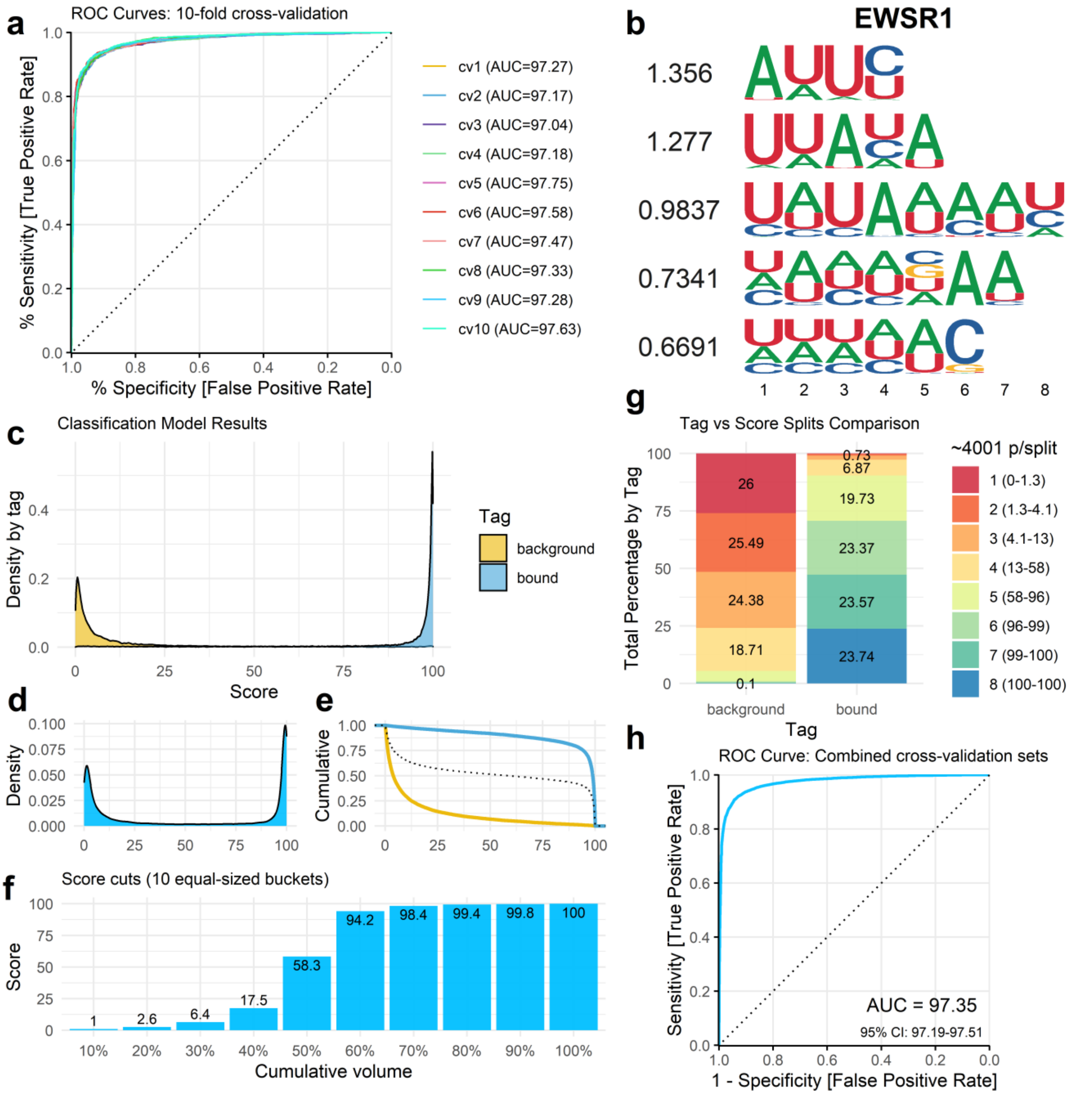

Figure S16 | DeepCLIP model characteristics for EWSR1. (a) Area under curve analysis of DeepCLIP models trained on EWSR1 PAR-CLIP data from Hoell et al. using 10-fold cross-validation. (b) Visualization of the CNN filters learned by the best performing model based on AUC. Score is equal to the mean information per base. (c-h) Visualizations of the combined model predictions of the 10-fold cross-validation. Scores are scaled to 0-100. (c) Density of background and bound prediction scores. (d) Combined density of prediction scores. (e) Cumulative predictive score of background and bound input sequences. (f) Barplot of cumulative scores of all input sequences. (g) Split prediction scores of background and bound input sequences. (h) Combined AUROC analysis using the pROC R package with DeLong estimation of 95% confidence interval.

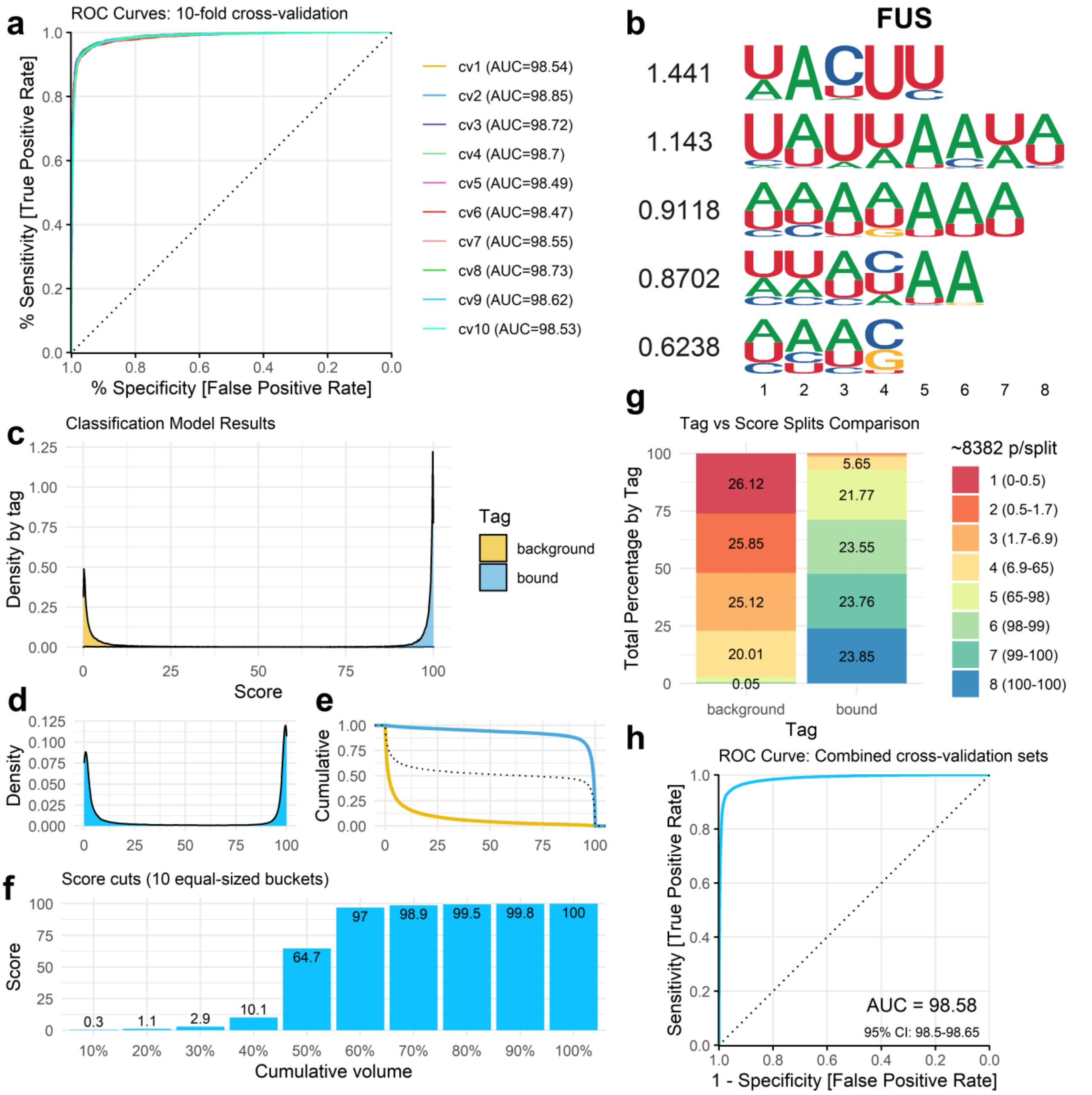

Figure S17 | DeepCLIP model characteristics for FUS. (a) Area under curve analysis of DeepCLIP models trained on FUS PAR-CLIP data from Hoell et al., using 10-fold cross-validation. (b) Visualization of the CNN filters learned by the best performing model based on AUC. Score is equal to the mean information per base. (c-h) Visualizations of the combined model predictions of the 10-fold cross-validation. Scores are scaled to 0-100. (c) Density of background and bound prediction scores. (d) Combined density of prediction scores. (e) Cumulative predictive score of background and bound input sequences. (f) Barplot of cumulative scores of all input sequences. (g) Split prediction scores of background and bound input sequences. (h) Combined AUROC analysis using the pROC R package with DeLong estimation of 95% confidence interval.

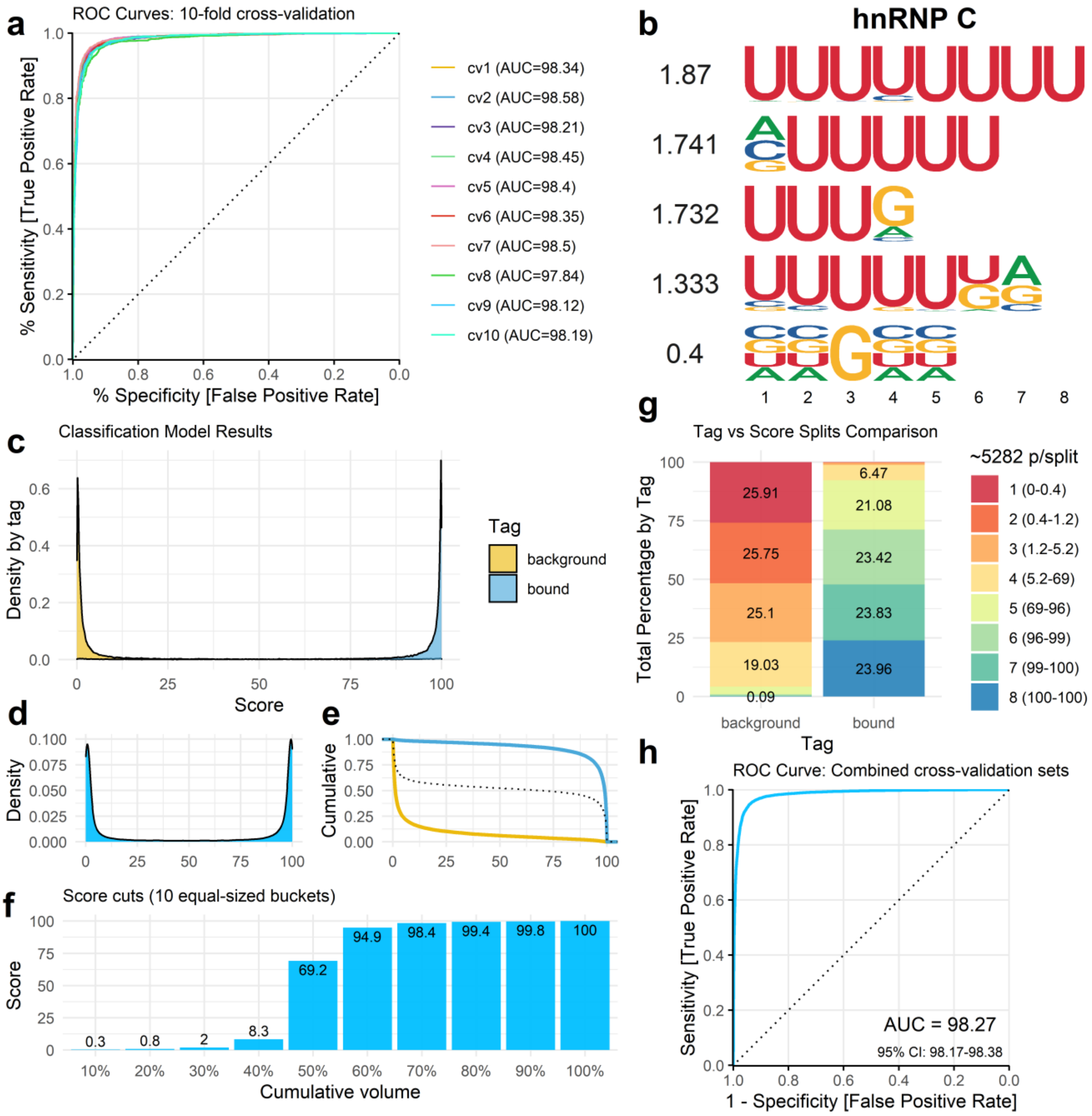

Figure S18 | DeepCLIP model characteristics for hnRNP C. (a) Area under curve analysis of DeepCLIP models trained on hnRNP C iCLIP data from König et al. using 10-fold cross-validation. (b) Visualization of the CNN filters learned by the best performing model based on AUC. Score is equal to the mean information per base. (c-h) Visualizations of the combined model predictions of the 10-fold cross-validation. Scores are scaled to 0-100. (c) Density of background and bound prediction scores. (d) Combined density of prediction scores. (e) Cumulative predictive score of background and bound input sequences. (f) Barplot of cumulative scores of all input sequences. (g) Split prediction scores of background and bound input sequences. (h) Combined AUROC analysis using the pROC R package with DeLong estimation of 95% confidence interval.

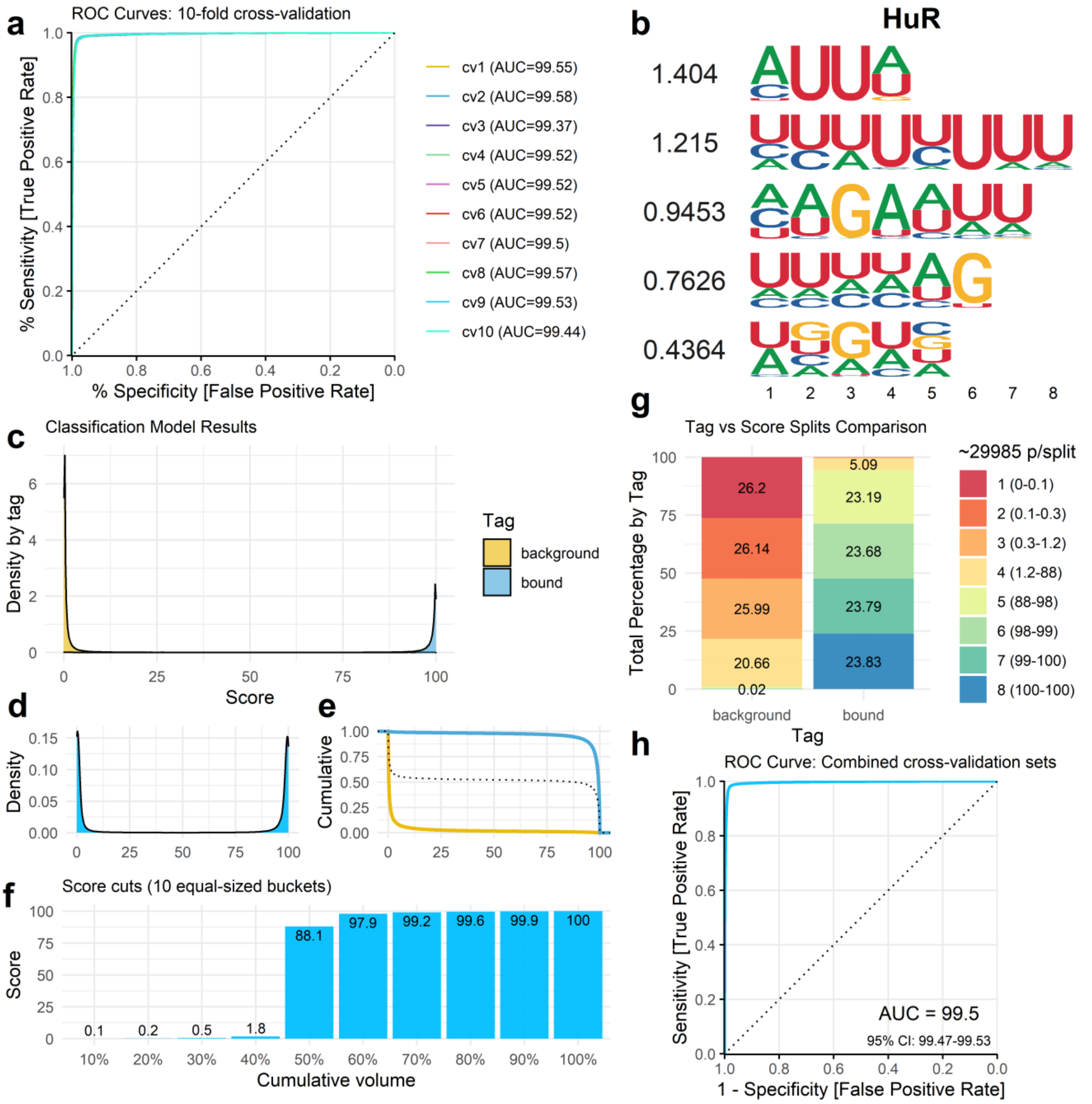

Figure S19 | DeepCLIP model characteristics for HuR. (a) Area under curve analysis of DeepCLIP models trained on HuR PAR-CLIP data from Mukherjee et al. using 10-fold cross-validation. (b) Visualization of the CNN filters learned by the best performing model based on AUC. Score is equal to the mean information per base. (c-h) Visualizations of the combined model predictions of the 10-fold cross-validation. Scores are scaled to 0-100. (c) Density of background and bound prediction scores. (d) Combined density of prediction scores. (e) Cumulative predictive score of background and bound input sequences. (f) Barplot of cumulative scores of all input sequences. (g) Split prediction scores of background and bound input sequences. (h) Combined AUROC analysis using the pROC R package with DeLong estimation of 95% confidence interval.

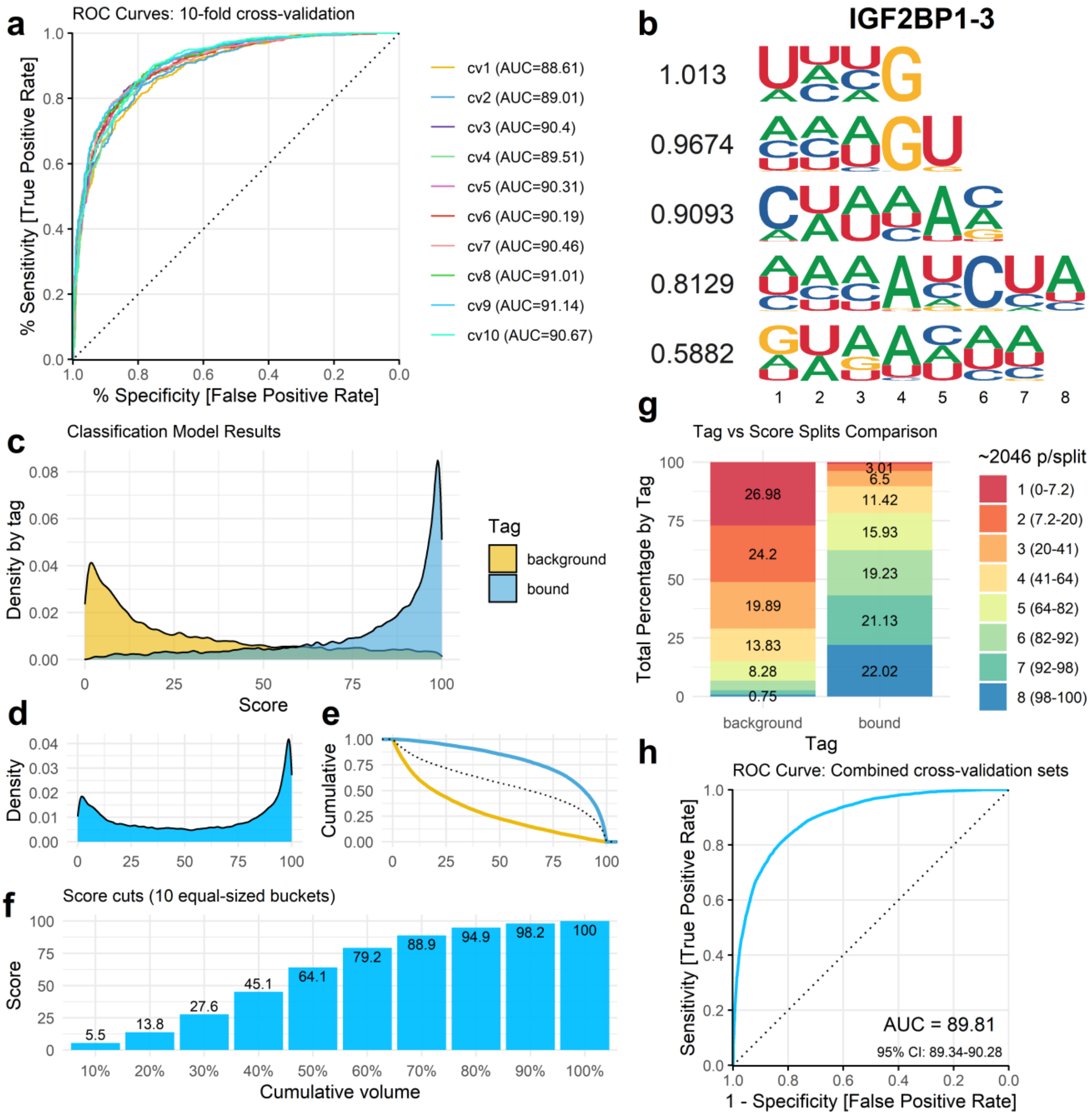

Figure S20 | DeepCLIP model characteristics for IGF2BP1-3. (a) Area under curve analysis of DeepCLIP models trained on IGF2BP1-3 PAR-CLIP data from Hafner et al. using 10-fold cross-validation. (b) Visualization of the CNN filters learned by the best performing model based on AUC. Score is equal to the mean information per base. (c-h) Visualizations of the combined model predictions of the 10-fold cross-validation. Scores are scaled to 0-100. (c) Density of background and bound prediction scores. (d) Combined density of prediction scores. (e) Cumulative predictive score of background and bound input sequences. (f) Barplot of cumulative scores of all input sequences. (g) Split prediction scores of background and bound input sequences. (h) Combined AUROC analysis using the pROC R package with DeLong estimation of 95% confidence interval.

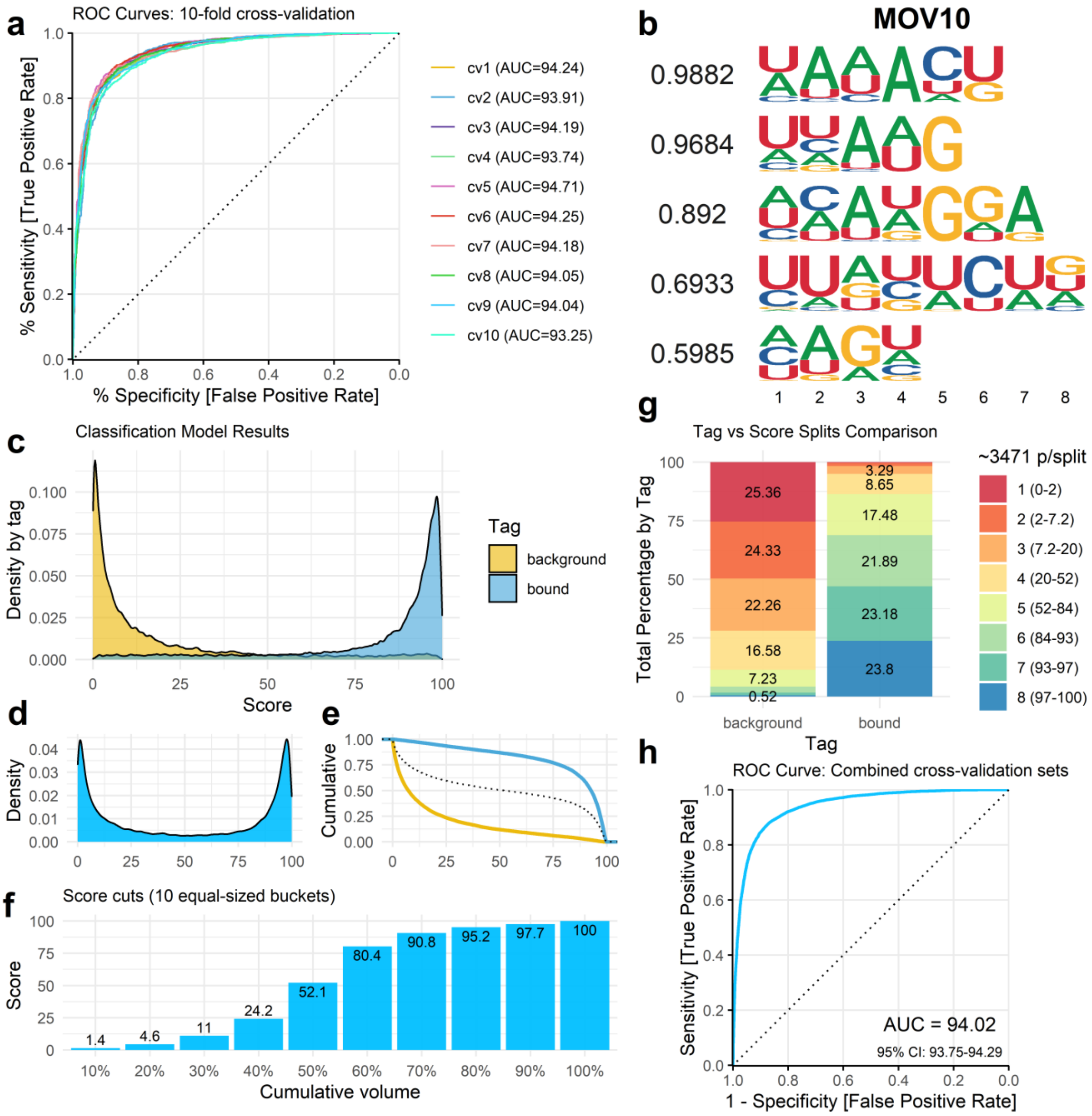

Figure S21 | DeepCLIP model characteristics for MOV10. (a) Area under curve analysis of DeepCLIP models trained on MOV10 PAR-CLIP data from Sievers et al. using 10-fold cross-validation. (b) Visualization of the CNN filters learned by the best performing model based on AUC. Score is equal to the mean information per base. (c-h) Visualizations of the combined model predictions of the 10-fold cross-validation. Scores are scaled to 0-100. (c) Density of background and bound prediction scores. (d) Combined density of prediction scores. (e) Cumulative predictive score of background and bound input sequences. (f) Barplot of cumulative scores of all input sequences. (g) Split prediction scores of background and bound input sequences. (h) Combined AUROC analysis using the pROC R package with DeLong estimation of 95% confidence interval.

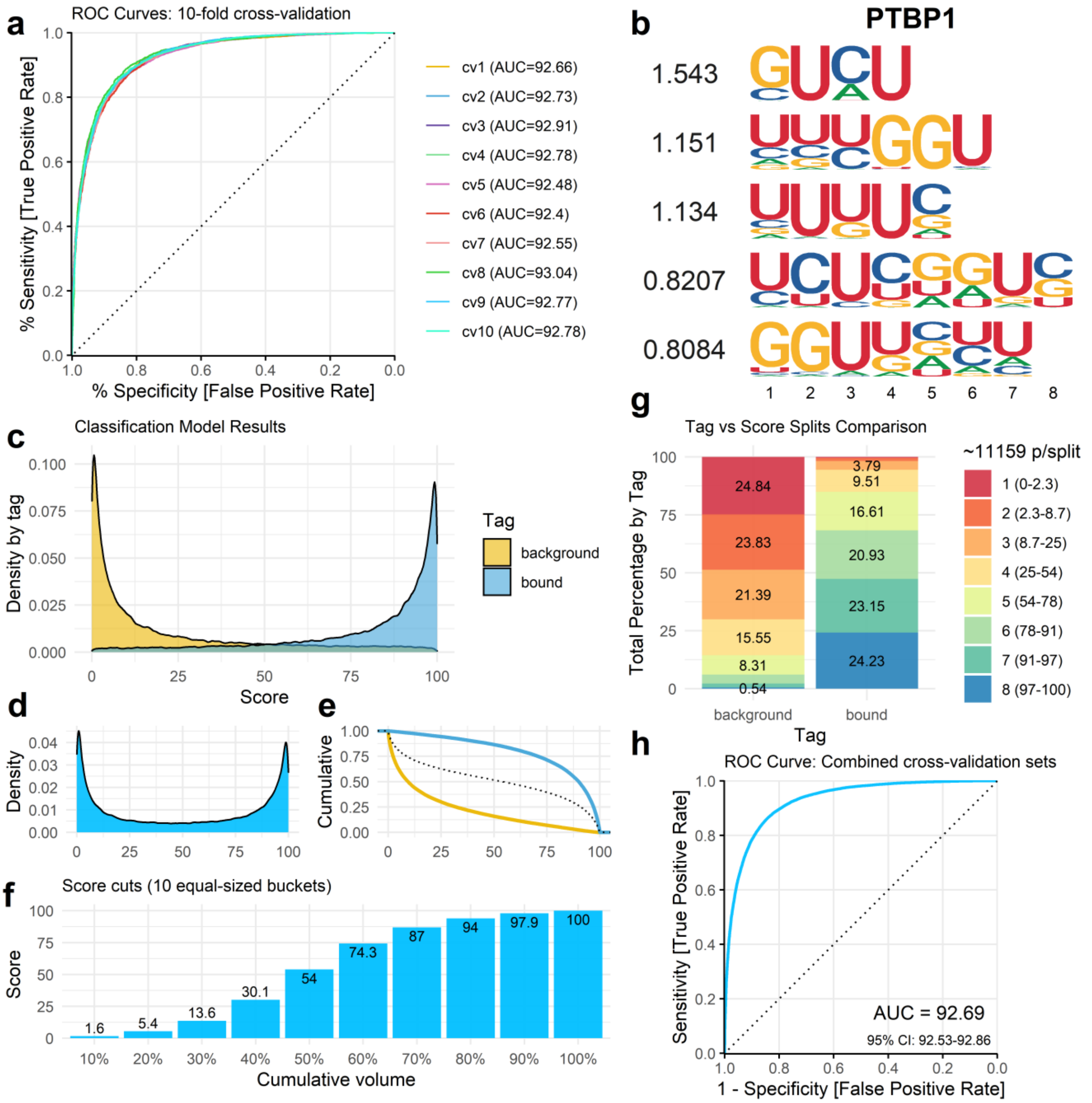

Figure S22 | DeepCLIP model characteristics for PTB1. (a) Area under curve analysis of DeepCLIP models trained on PTB1 HITS-CLIP data from Xue et al. using 10-fold cross-validation. (b) Visualization of the CNN filters learned by the best performing model based on AUC. Score is equal to the mean information per base. (c-h) Visualizations of the combined model predictions of the 10-fold cross-validation. Scores are scaled to 0-100. (c) Density of background and bound prediction scores. (d) Combined density of prediction scores. (e) Cumulative predictive score of background and bound input sequences. (f) Barplot of cumulative scores of all input sequences. (g) Split prediction scores of background and bound input sequences. (h) Combined AUROC analysis using the pROC R package with DeLong estimation of 95% confidence interval.

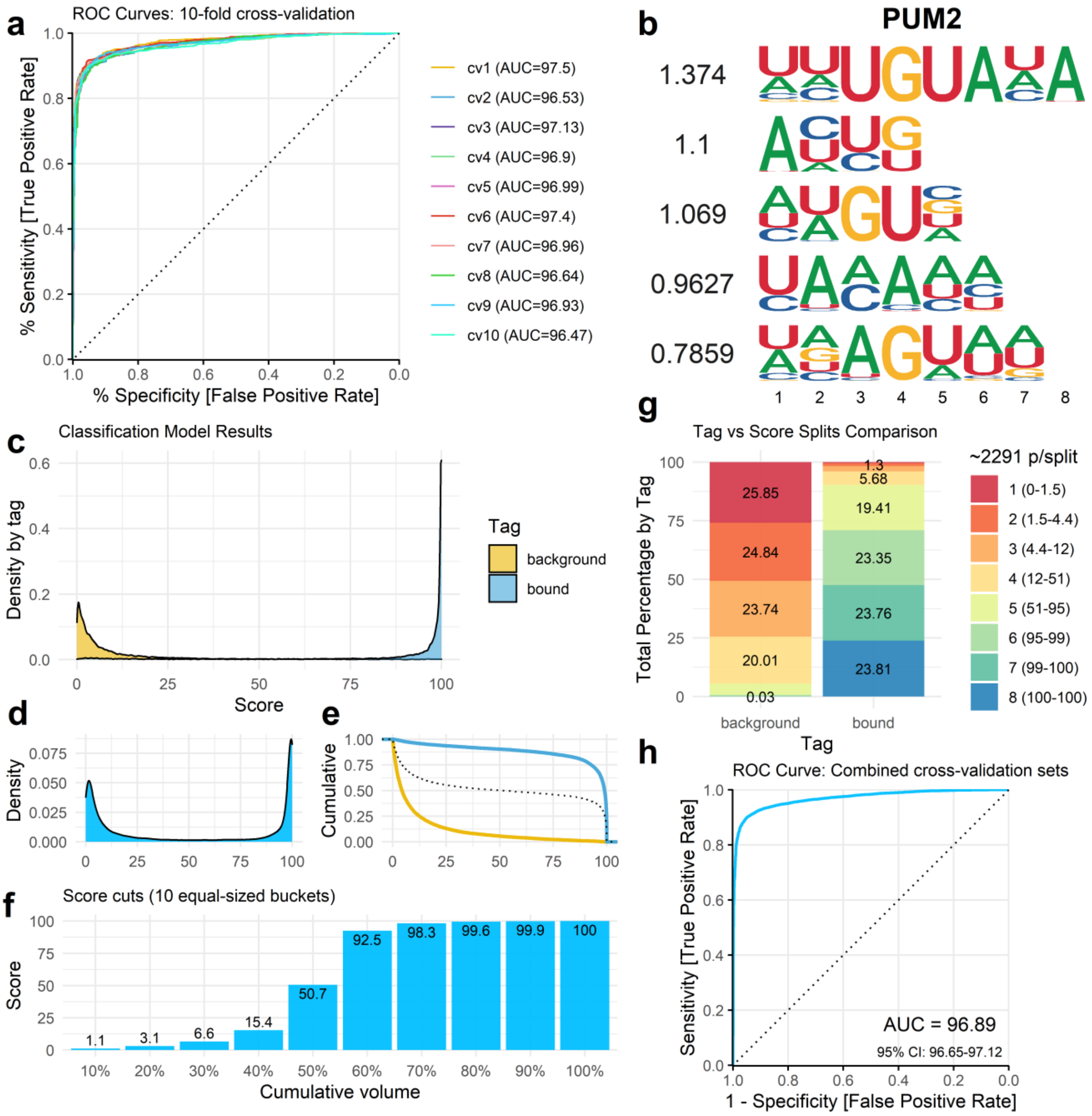

Figure S23 | DeepCLIP model characteristics for PUM2. (a) Area under curve analysis of DeepCLIP models trained on PUM2 PAR-CLIP data from Hafner et al. using 10-fold cross-validation. (b) Visualization of the CNN filters learned by the best performing model based on AUC. Score is equal to the mean information per base. (c-h) Visualizations of the combined model predictions of the 10-fold cross-validation. Scores are scaled to 0-100. (c) Density of background and bound prediction scores. (d) Combined density of prediction scores. (e) Cumulative predictive score of background and bound input sequences. (f) Barplot of cumulative scores of all input sequences. (g) Split prediction scores of background and bound input sequences. (h) Combined AUROC analysis using the pROC R package with DeLong estimation of 95% confidence interval.

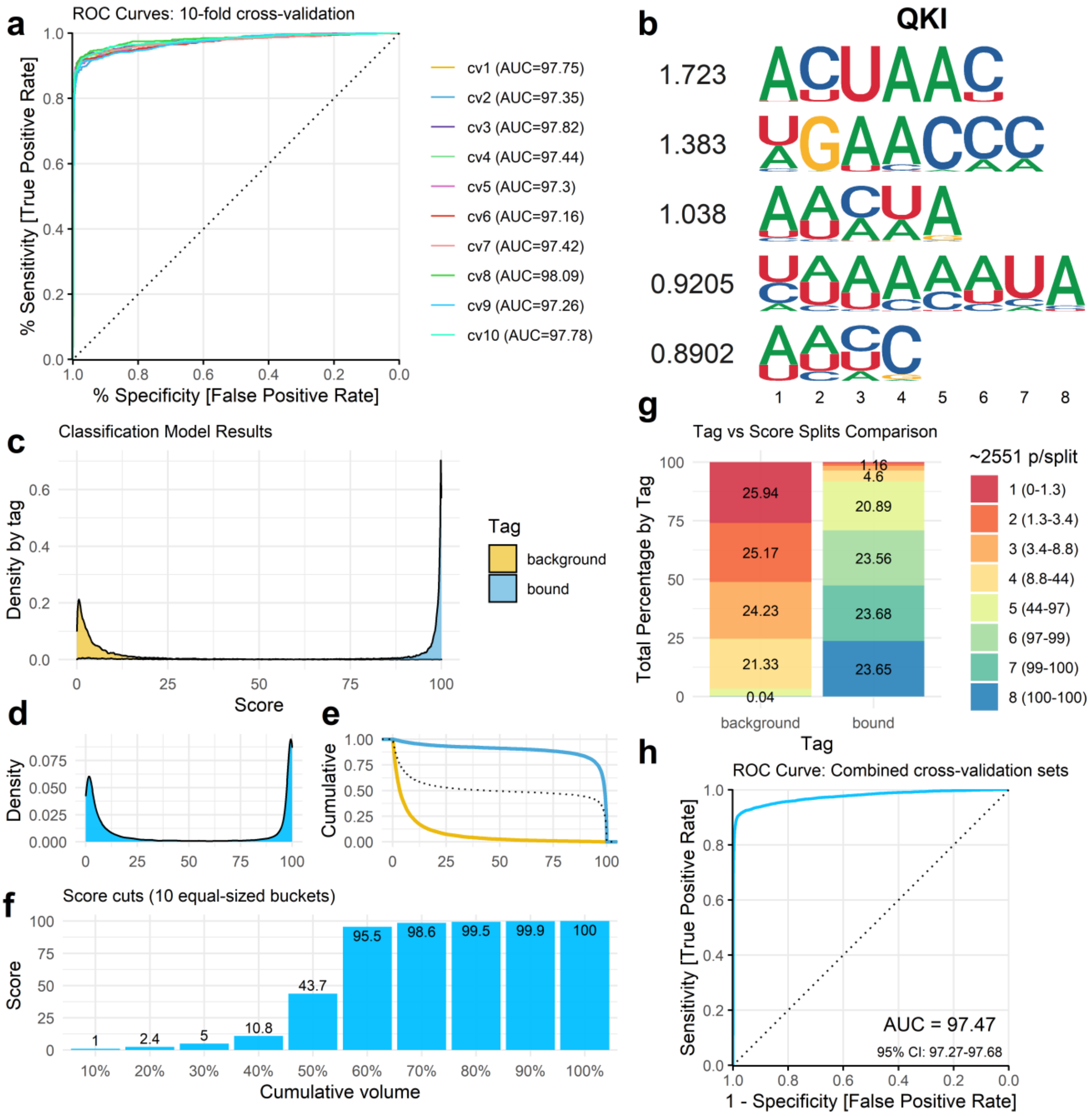

Figure S24 | DeepCLIP model characteristics for QKI. (a) Area under curve analysis of DeepCLIP models trained on QKI PAR-CLIP data from Hafner et al. using 10-fold cross-validation. (b) Visualization of the CNN filters learned by the best performing model based on AUC. Score is equal to the mean information per base. (c-h) Visualizations of the combined model predictions of the 10-fold cross-validation. Scores are scaled to 0-100. (c) Density of background and bound prediction scores. (d) Combined density of prediction scores. (e) Cumulative predictive score of background and bound input sequences. (f) Barplot of cumulative scores of all input sequences. (g) Split prediction scores of background and bound input sequences. (h) Combined AUROC analysis using the pROC R package with DeLong estimation of 95% confidence interval.

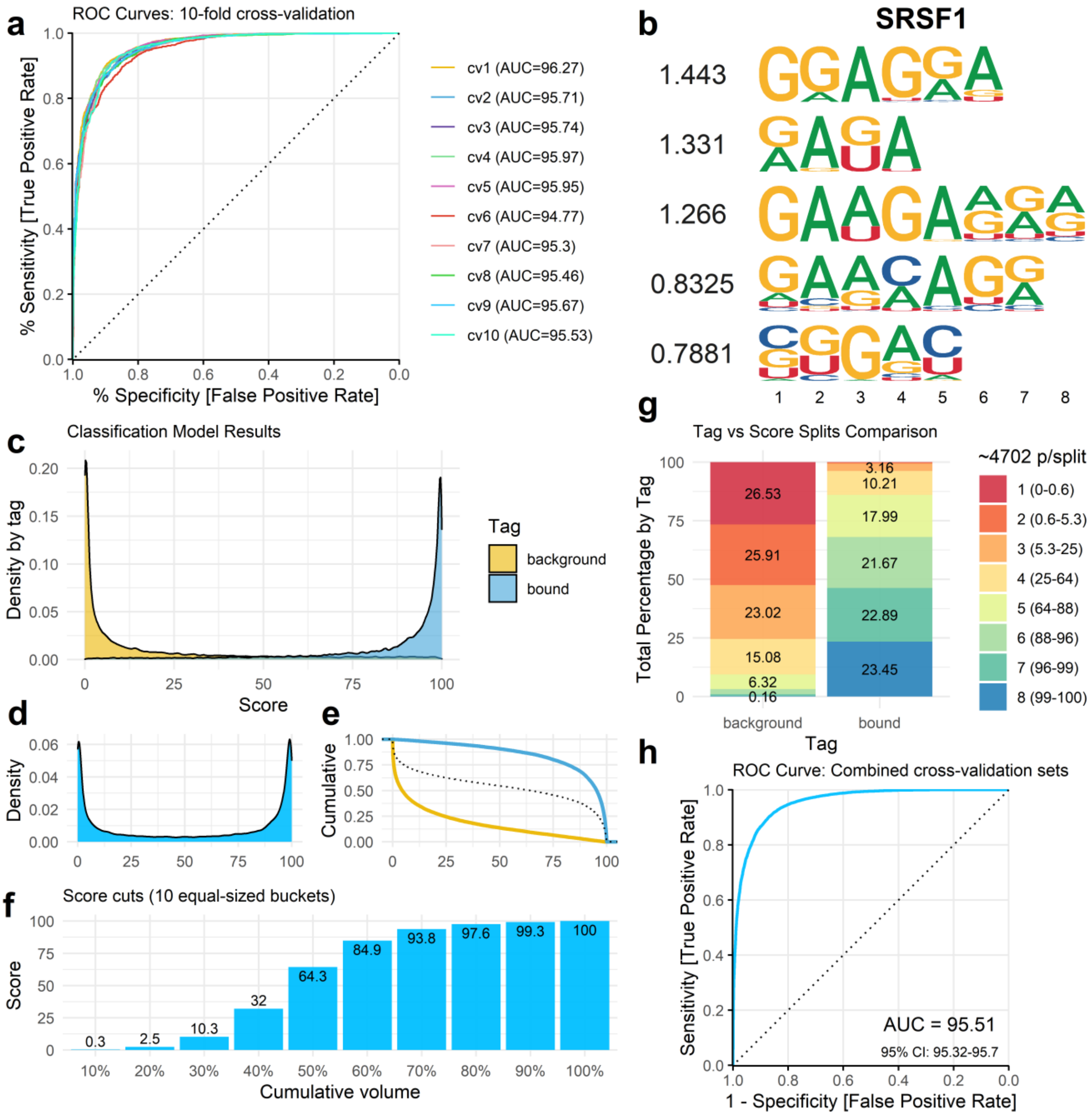

Figure S25 | DeepCLIP model characteristics for SRSF1. (a) Area under curve analysis of DeepCLIP models trained on SRSF1 HITS-CLIP data from Sanford et al. using 10-fold cross-validation. (b) Visualization of the CNN filters learned by the best performing model based on AUC. Score is equal to the mean information per base. (c-h) Visualizations of the combined model predictions of the 10-fold cross-validation. Scores are scaled to 0-100. (c) Density of background and bound prediction scores. (d) Combined density of prediction scores. (e) Cumulative predictive score of background and bound input sequences. (f) Barplot of cumulative scores of all input sequences. (g) Split prediction scores of background and bound input sequences. (h) Combined AUROC analysis using the pROC R package with DeLong estimation of 95% confidence interval.

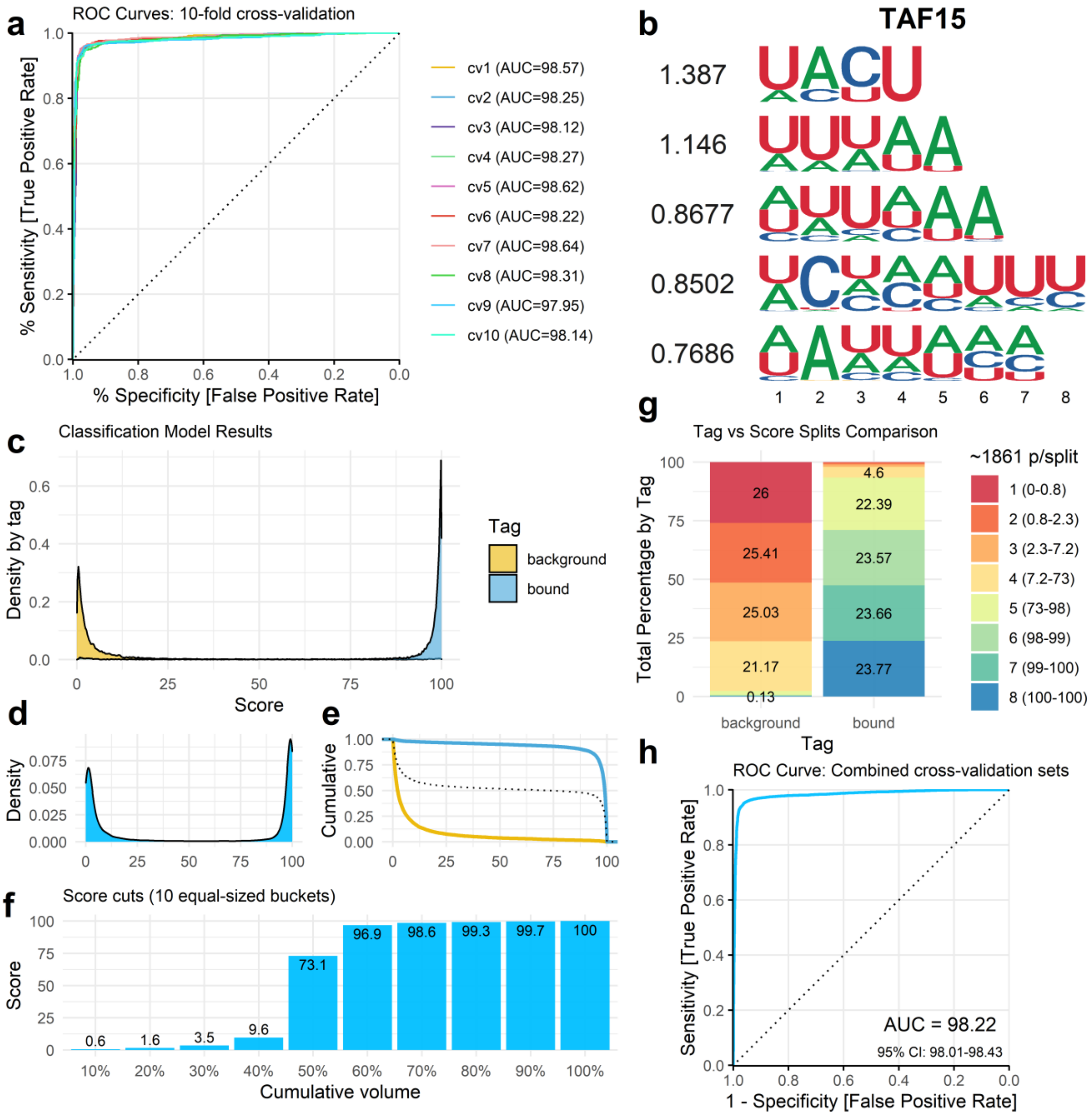

Figure S26 | DeepCLIP model characteristics for TAF15. (a) Area under curve analysis of DeepCLIP models trained on TAF15 PAR-CLIP data from Hoell et al. using 10-fold cross-validation. (b) Visualization of the CNN filters learned by the best performing model based on AUC. Score is equal to the mean information per base. (c-h) Visualizations of the combined model predictions of the 10-fold cross-validation. Scores are scaled to 0-100. (c) Density of background and bound prediction scores. (d) Combined density of prediction scores. (e) Cumulative predictive score of background and bound input sequences. (f) Barplot of cumulative scores of all input sequences. (g) Split prediction scores of background and bound input sequences. (h) Combined AUROC analysis using the pROC R package with DeLong estimation of 95% confidence interval.

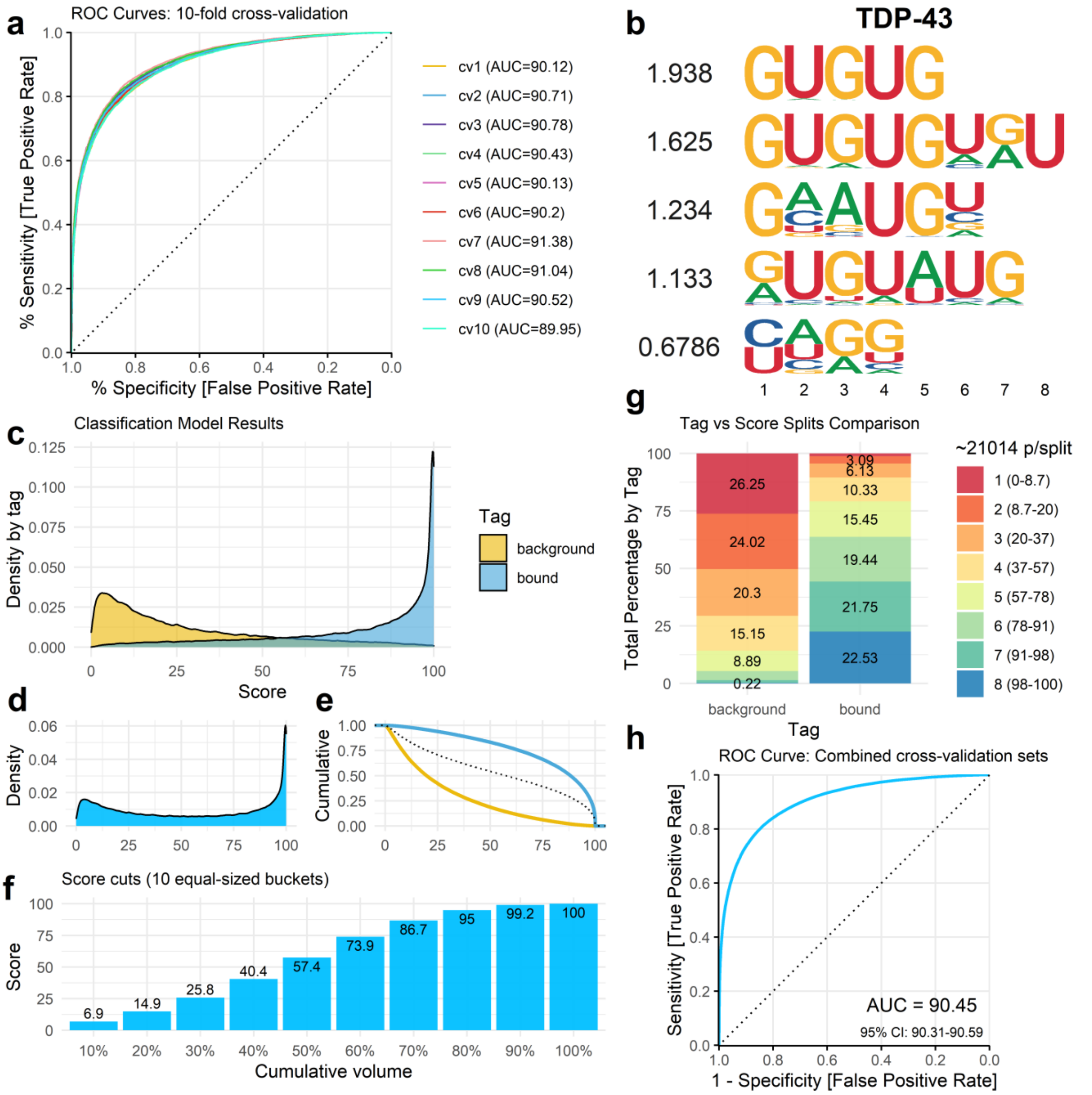

Figure S27 | DeepCLIP model characteristics for TDP-43. (a) Area under curve analysis of DeepCLIP models trained on TDP-43 iCLIP data from Tollervey et al. using 10-fold cross-validation. (b) Visualization of the CNN filters learned by the best performing model based on AUC. Score is equal to the mean information per base. (c-h) Visualizations of the combined model predictions of the 10-fold cross-validation. Scores are scaled to 0-100. (c) Density of background and bound prediction scores. (d) Combined density of prediction scores. (e) Cumulative predictive score of background and bound input sequences. (f) Barplot of cumulative scores of all input sequences. (g) Split prediction scores of background and bound input sequences. (h) Combined AUROC analysis using the pROC R package with DeLong estimation of 95% confidence interval.

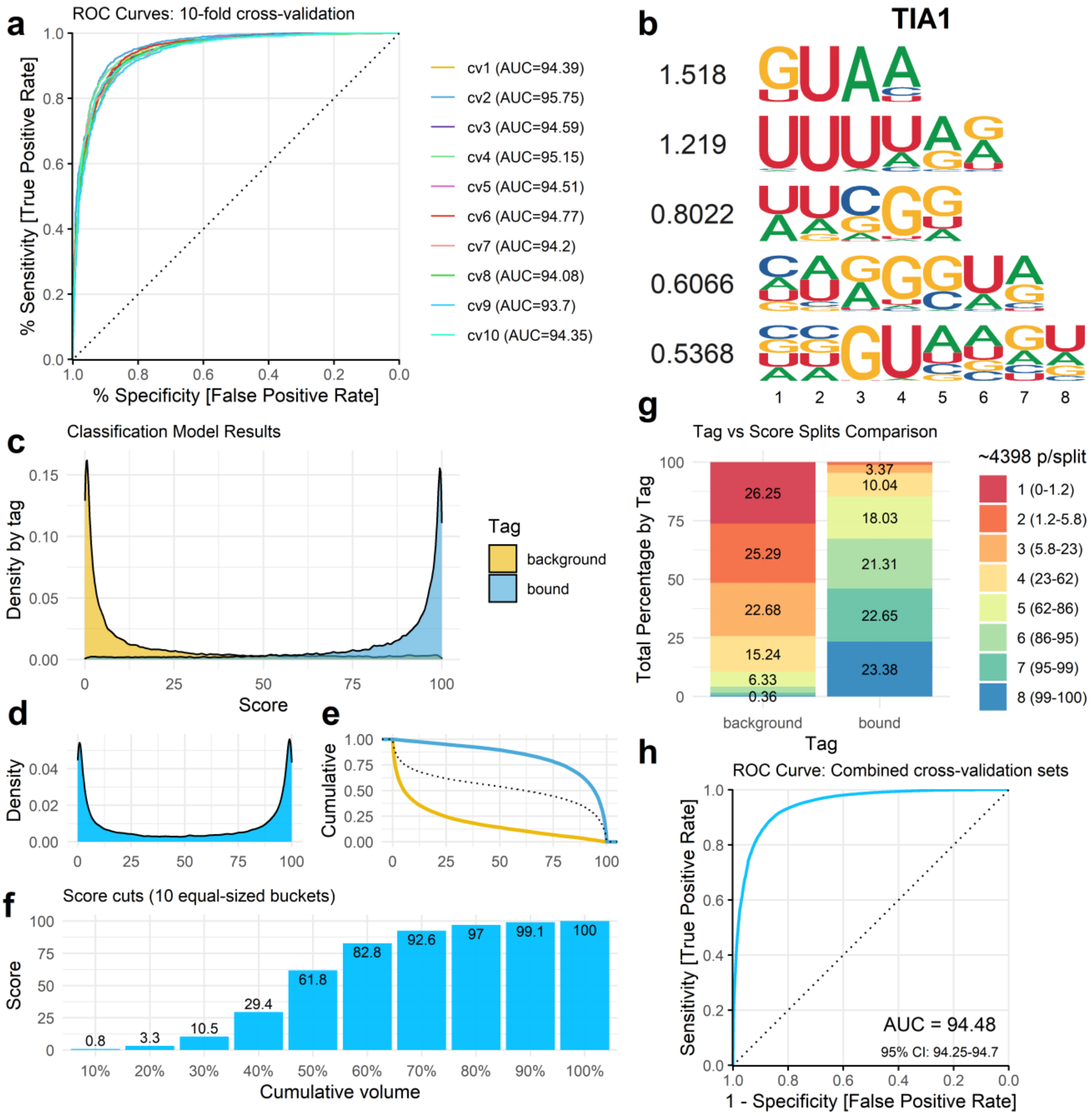

Figure S28 | DeepCLIP model characteristics for TIA1. (a) Area under curve analysis of DeepCLIP models trained on TIA1 iCLIP data from Wang et al. using 10-fold cross-validation. (b) Visualization of the CNN filters learned by the best performing model based on AUC. Score is equal to the mean information per base. (c-h) Visualizations of the combined model predictions of the 10-fold cross-validation. Scores are scaled to 0-100. (c) Density of background and bound prediction scores. (d) Combined density of prediction scores. (e) Cumulative predictive score of background and bound input sequences. (f) Barplot of cumulative scores of all input sequences. (g) Split prediction scores of background and bound input sequences. (h) Combined AUROC analysis using the pROC R package with DeLong estimation of 95% confidence interval.

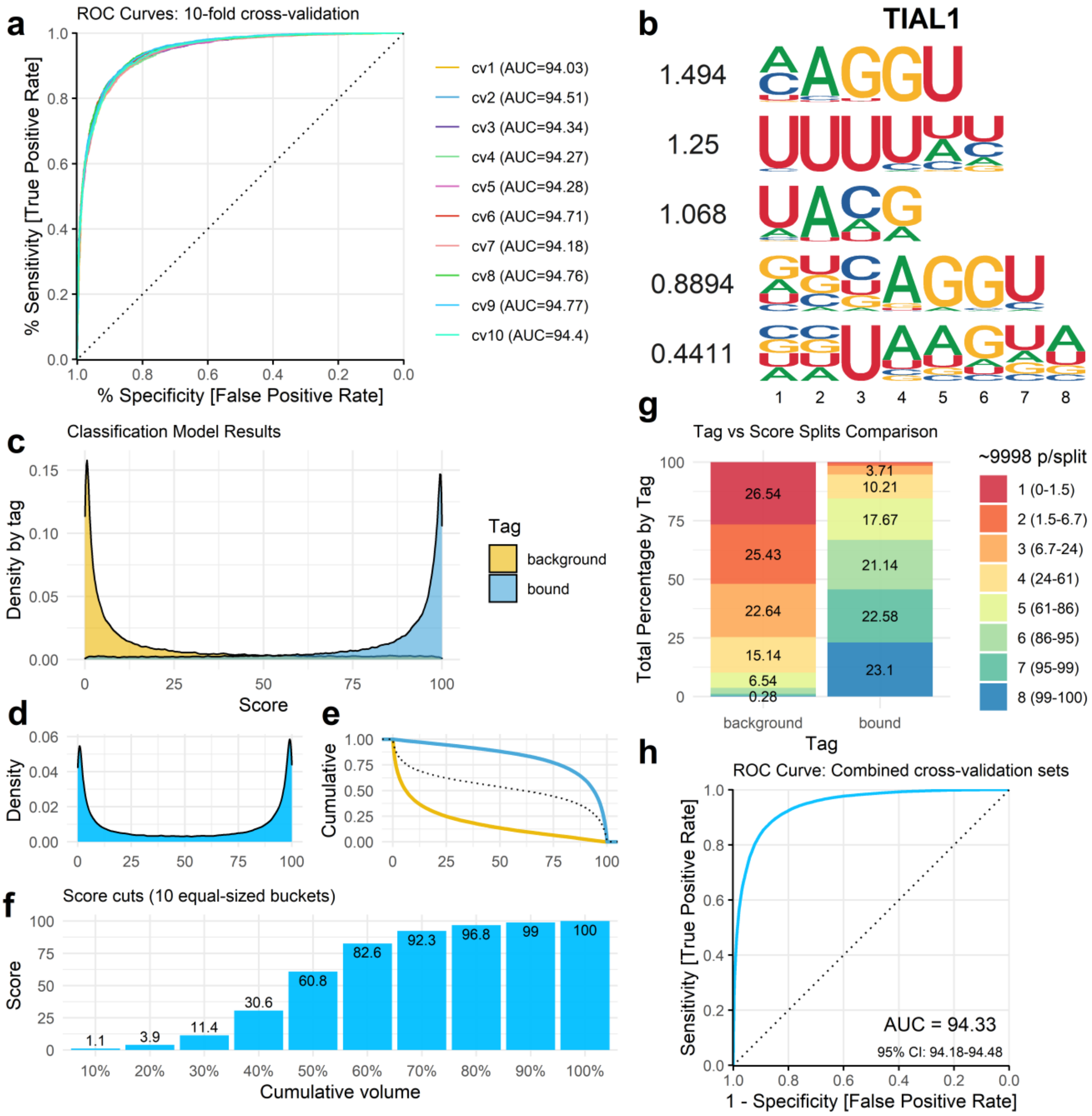

Figure S29 | DeepCLIP model characteristics for TIAL1. (a) Area under curve analysis of DeepCLIP models trained on TIAL1 PAR-CLIP data from Wang et al. using 10-fold cross-validation. (b) Visualization of the CNN filters learned by the best performing model based on AUC. Score is equal to the mean information per base. (c-h) Visualizations of the combined model predictions of the 10-fold cross-validation. Scores are scaled to 0-100. (c) Density of background and bound prediction scores. (d) Combined density of prediction scores. (e) Cumulative predictive score of background and bound input sequences. (f) Barplot of cumulative scores of all input sequences. (g) Split prediction scores of background and bound input sequences. (h) Combined AUROC analysis using the pROC R package with DeLong estimation of 95% confidence interval.

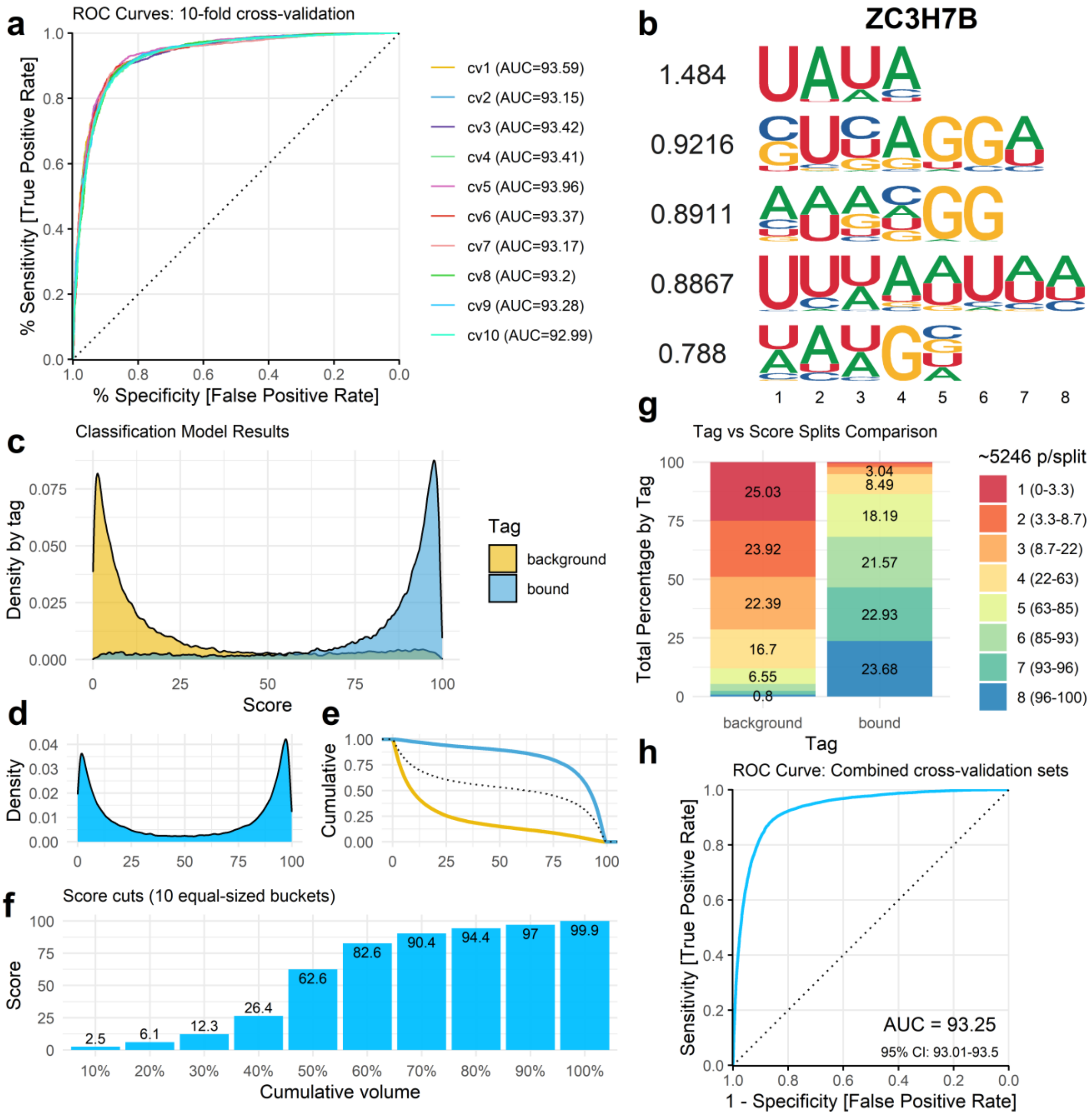

Figure S30 | DeepCLIP model characteristics for ZC3H7B. (a) Area under curve analysis of DeepCLIP models trained on ZC3H7B PAR-CLIP data from Baltz et al. using 10-fold cross-validation. (b) Visualization of the CNN filters learned by the best performing model based on AUC. Score is equal to the mean information per base. (c-h) Visualizations of the combined model predictions of the 10-fold cross-validation. Scores are scaled to 0-100. (c) Density of background and bound prediction scores. (d) Combined density of prediction scores. (e) Cumulative predictive score of background and bound input sequences. (f) Barplot of cumulative scores of all input sequences. (g) Split prediction scores of background and bound input sequences. (h) Combined AUROC analysis using the pROC R package with DeLong estimation of 95% confidence interval.

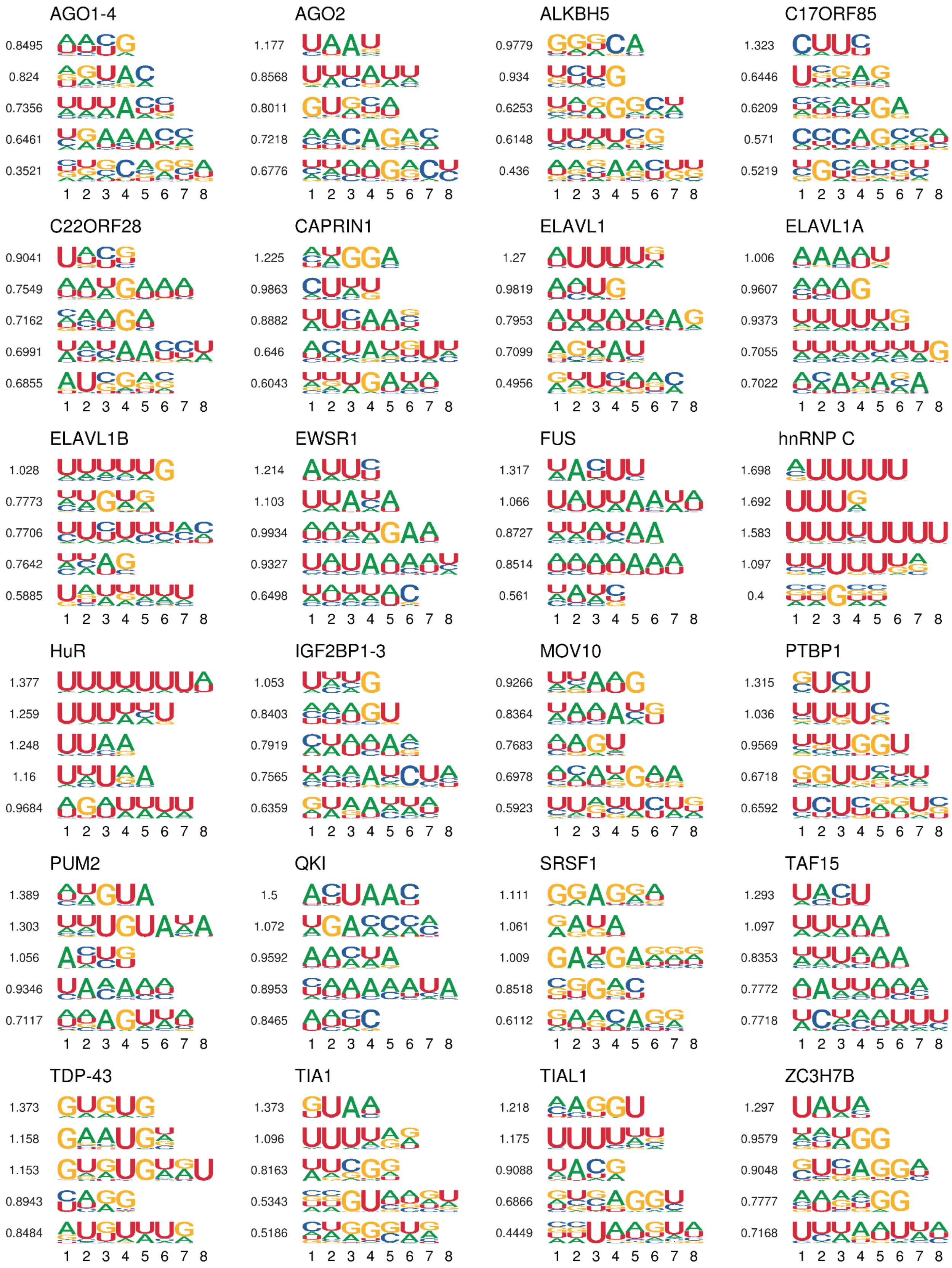

Figure S31 | Alternative CNN filter extraction. CNN filters produced from the sequences among the total training set data, both positives and negatives, scoring above 0.5.

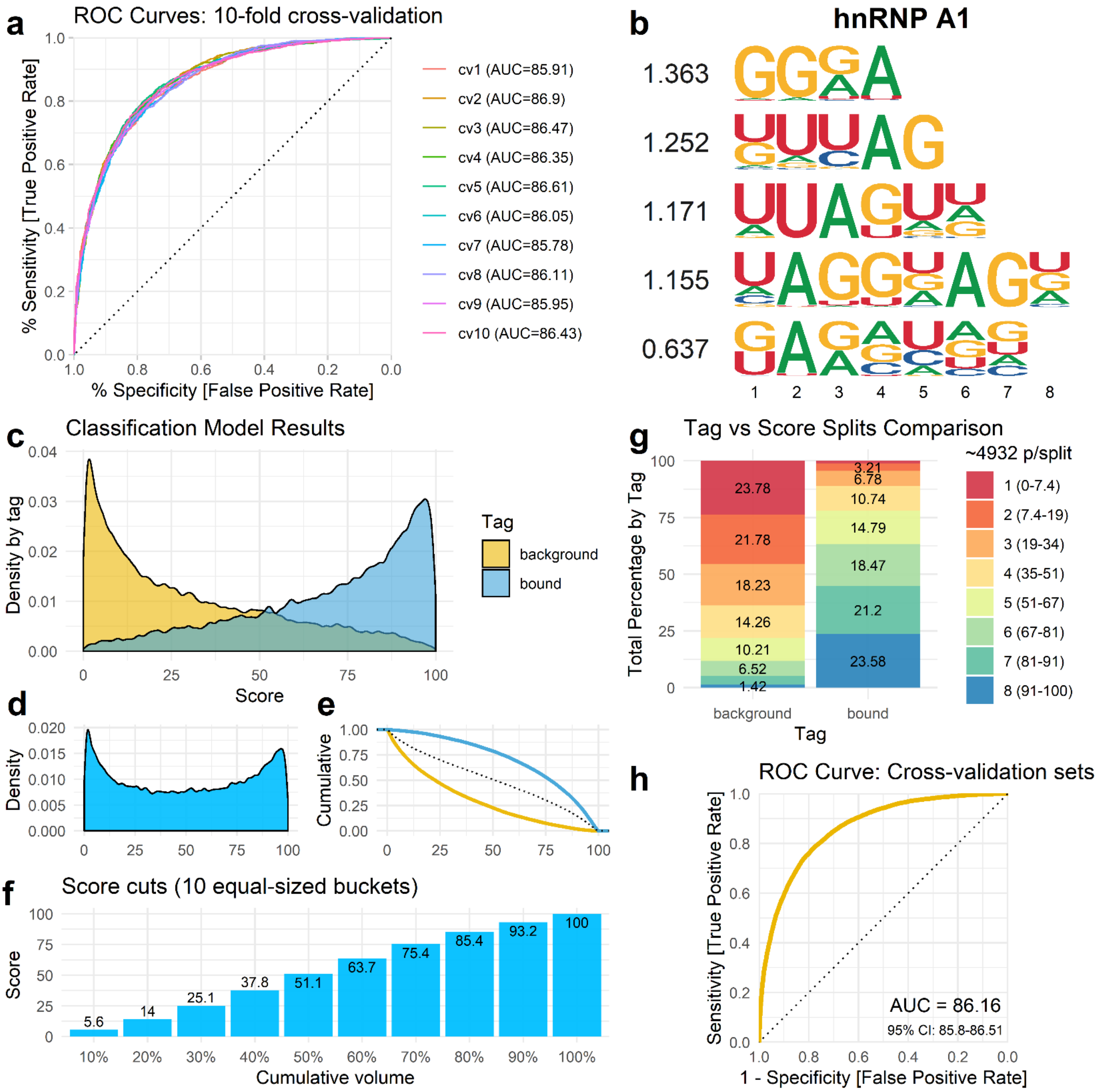

Figure S32 | DeepCLIP model characteristics for hnRNP A1. (a) Area under curve analysis of DeepCLIP models trained on hnRNP A1 iCLIP data from Bruun et al. using 10-fold cross-validation. (b) Visualization of the CNN filters learned by the best performing model based on AUC. Score is equal to the mean information per base. (c-h) Visualizations of the combined model predictions of the 10-fold cross-validation. Scores are scaled to 0-100. (c) Density of background and bound prediction scores. (d) Combined density of prediction scores. (e) Cumulative predictive score of background and bound input sequences. (f) Barplot of cumulative scores of all input sequences. (g) Split prediction scores of background and bound input sequences. (h) Combined AUROC analysis using the pROC R package with DeLong estimation of 95% confidence interval.

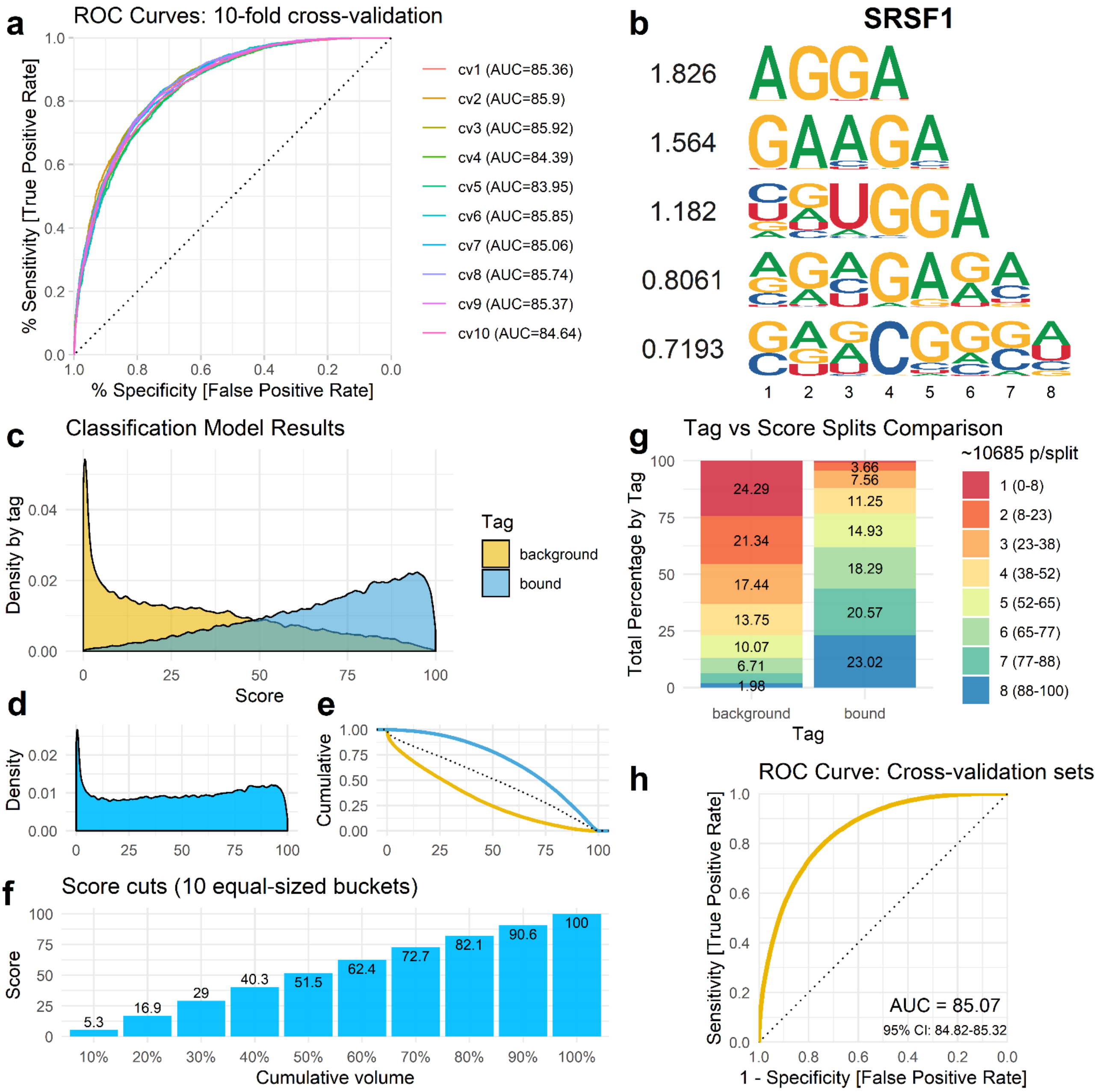

Figure S33 | DeepCLIP model characteristics for SRSF1 (eCLIP). (a) Area under curve analysis of DeepCLIP models trained on SRSF1 eCLIP data from Van Nostrand et al using 10-fold cross-validation. (b) Visualization of the CNN filters learned by the best performing model based on AUC. Score is equal to the mean information per base. (c-h) Visualizations of the combined model predictions of the 10-fold cross-validation. Scores are scaled to 0-100. (c) Density of background and bound prediction scores. (d) Combined density of prediction scores. (e) Cumulative predictive score of background and bound input sequences. (f) Barplot of cumulative scores of all input sequences. (g) Split prediction scores of background and bound input sequences. (h) Combined AUROC analysis using the pROC R package with DeLong estimation of 95% confidence interval.

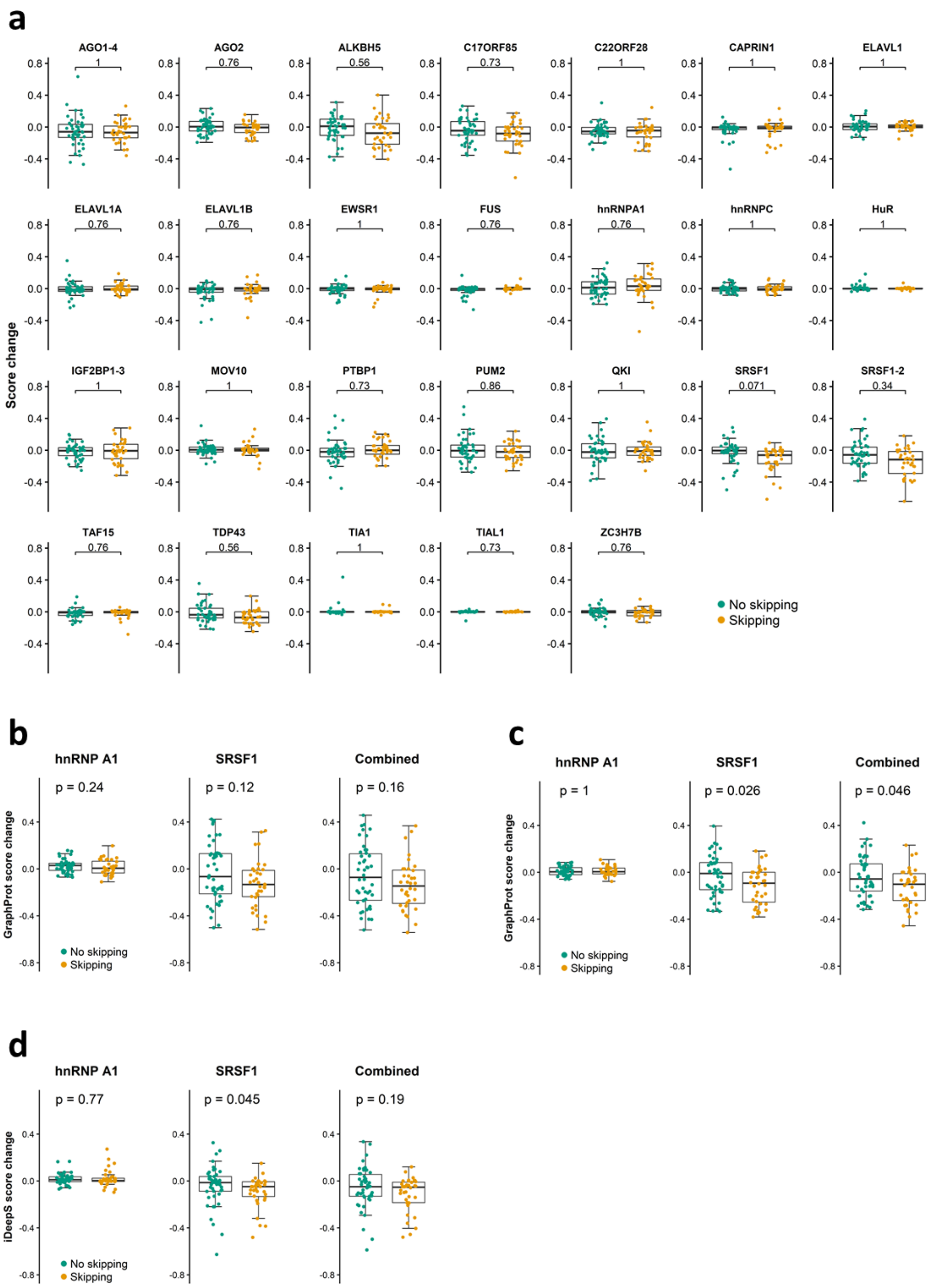

Figure S34 | Boxplots of Raponi dataset using previously trained models. a) Boxplots of Raponi dataset using all models in the GraphProt benchmark dataset, and the new SRSF1 and hnRNP A1 model used in this study. B) Analysis of Raponi et al dataset with GraphProt models trained on the SRSF1 eCLIP and hnRNP A1 iCLIP data used to train DeepCLIP models in this study., padded with 150 nt genomic context on each side, according to GraphProt guidelines.. Input sequences for Raponi et al analysis were 15 nt padded with 150 nt genomic sequence on each side of the 15 nt viewpoint. C) Same as (b), but with 75nt viewpoint. D) Analysis of Raponi et al dataset with iDeepS models generated from 101nt input training data corresponding to the same sites used to train SRSF1 and hnRNP A1 DeepCLIP and GraphProt models. In all charts are shown boxplot of the score change between wt and mutation leading either to skipping (yellow) or no skipping (green).

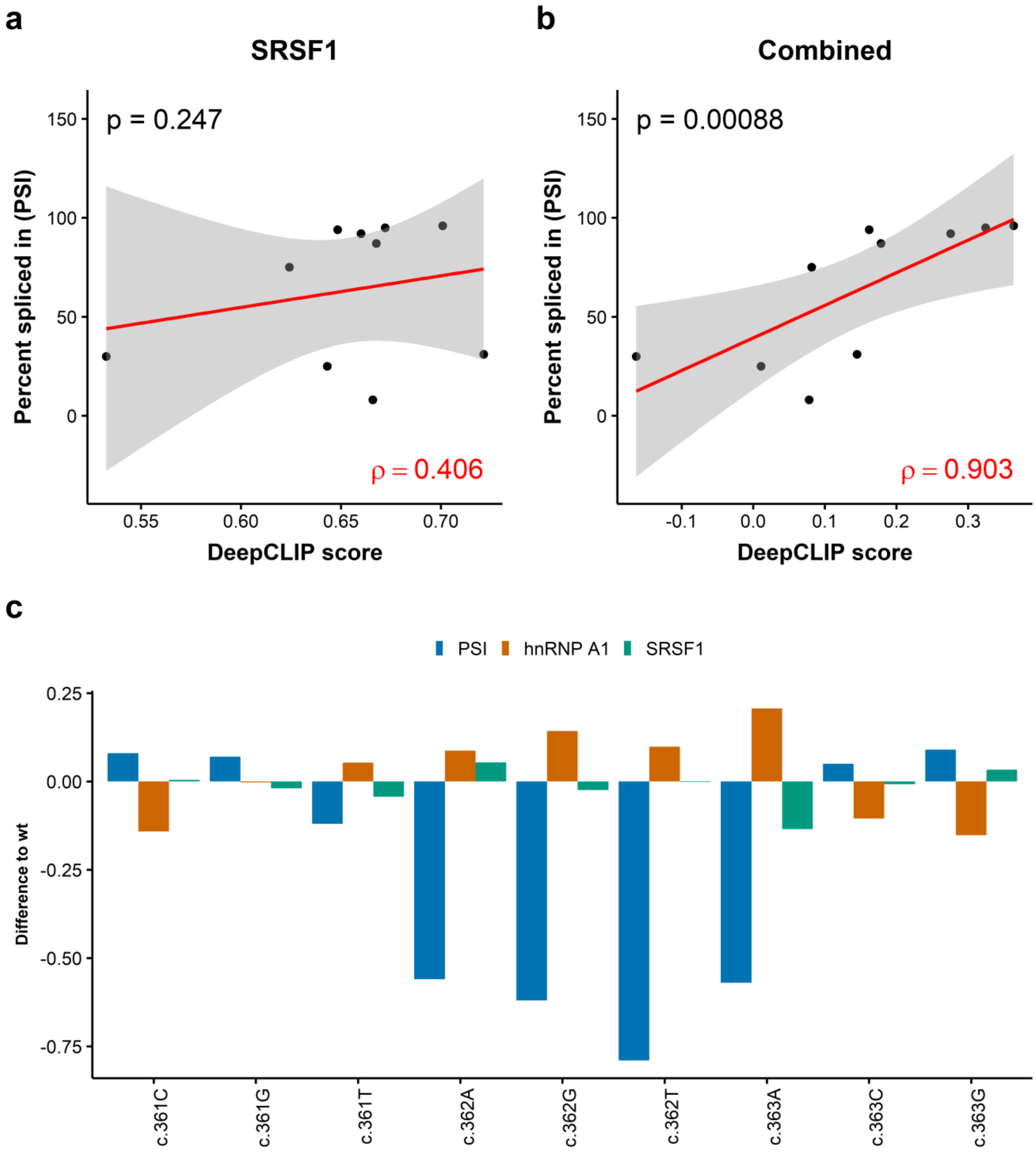

Figure S35 | DeepCLIP predictions of *ACADM* exon 5 mutations using SRSF1 (HITS-CLIP) model. (a) Scatter plot of *ACADM* exon 5 minigene percent spliced in (PSI) values and DeepCLIP SRSF1 score with linear regression (red line) and 95% confidence interval (shaded area). (b) Same as (a) but with the DeepCLIP hnRNP A1 score subtracted from the SRSF1 score. Spearman's rho is indicated in red for both plots in (a-b). (c) Barplot of the change relative to the wt of exon inclusion levels (blue), hnRNP A1 DeepCLIP scores (brown) and SRSF1 HITS-CLIP DeepCLIP model scores (green). The hnRNP A1 results are the same as presented in figure 5 but are included here for reference.

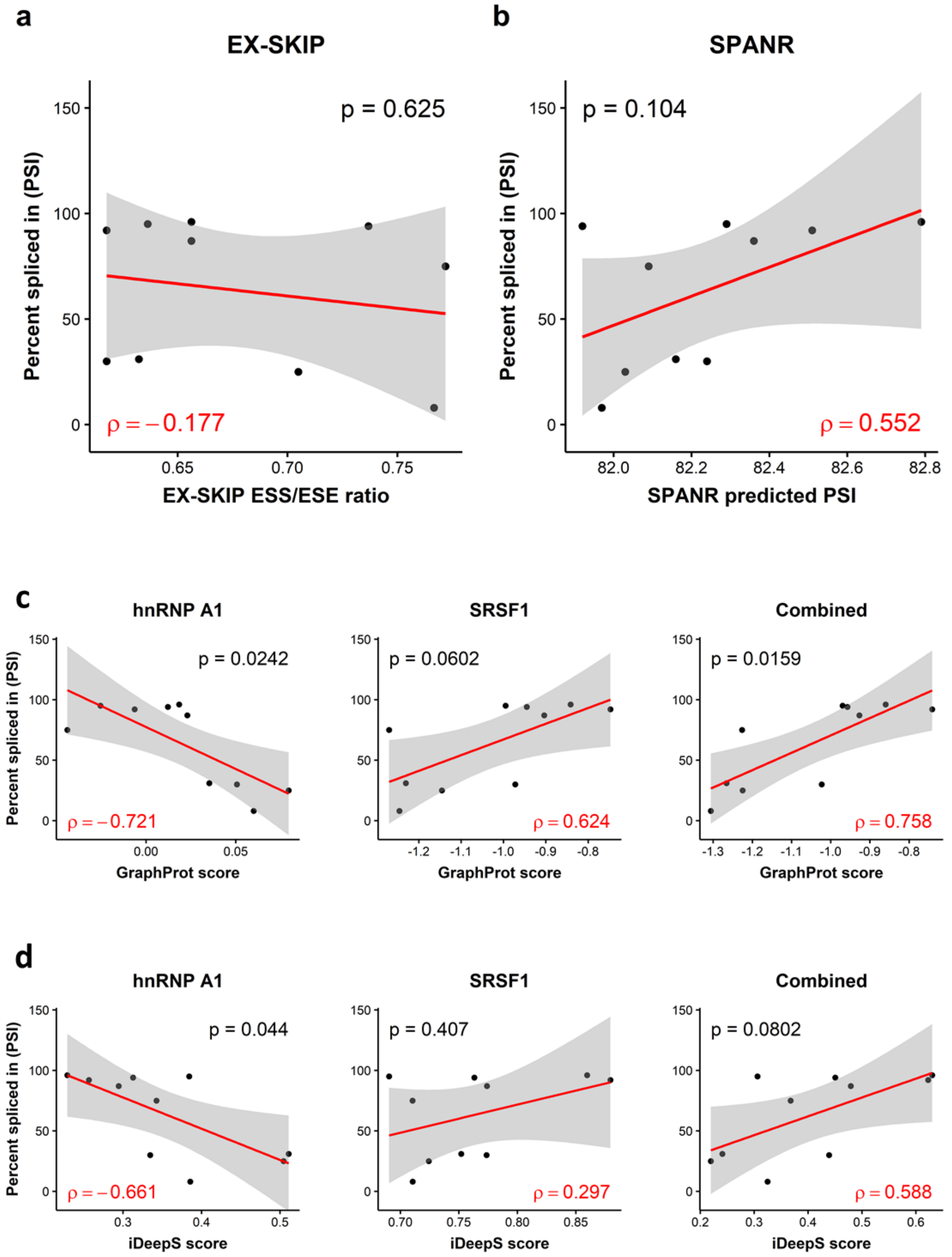

Figure S36 | Correlation between observed exon skipping and exon skipping model predictions. (a) Scatter plot of exon inclusion levels vs EX-SKIP predictions. (b) Scatter plot of the exon inclusions levels vs the SPANR prediction exon inclusion levels. (c) Scatter plots of exon inclusion levels vs predictions of GraphProt hnRNP A1 model (left), GraphProt SRSF1 model (middle) and GraphProt hnRNP A1 and SRSF1 models combined (right). The combined change in GraphProt scores is obtained by subtracting the hnRNP A1 scores from the SRSF1 scores (d) same as (c) but with iDeepS hnRNP A1 and SRSF1 models. Spearman's rho is indicated in red for both plots in (a-d).

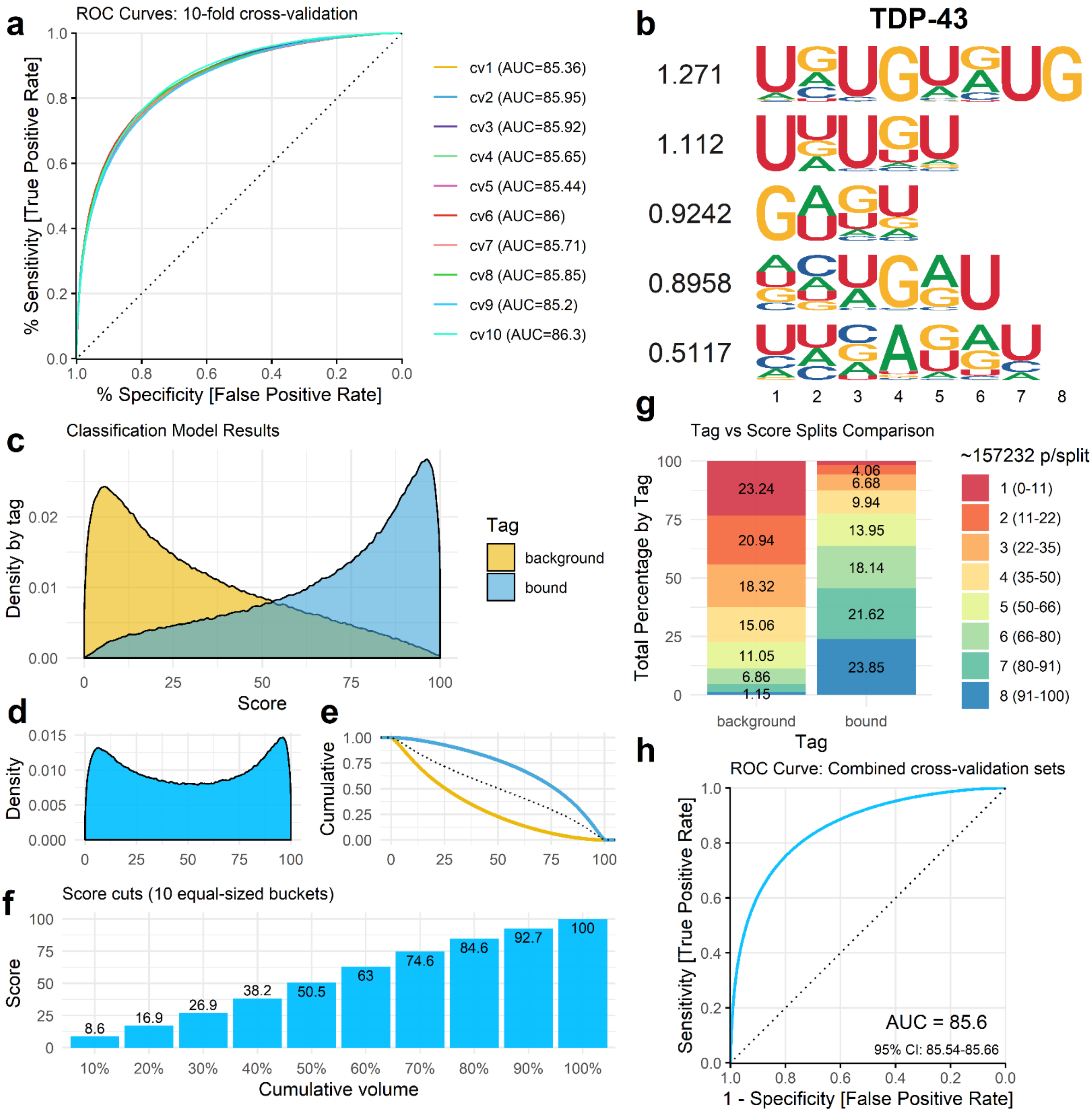

Figure S37 | DeepCLIP model characteristics for TDP43. (a) Area under curve analysis of DeepCLIP models trained on TDP43 binding sites from the POSTAR2database using 10-fold cross-validation. (b) Visualization of the CNN filters learned by the best performing model based on AUC. Score is equal to the mean information per base. (c-h) Visualizations of the combined model predictions of the 10-fold cross-validation. Scores are scaled to 0-100. (c) Density of background and bound prediction scores. (d) Combined density of prediction scores. (e) Cumulative predictive score of background and bound input sequences. (f) Barplot of cumulative scores of all input sequences. (g) Split prediction scores of background and bound input sequences. (h) Combined AUROC analysis using the pROC R package with DeLong estimation of 95% confidence interval.

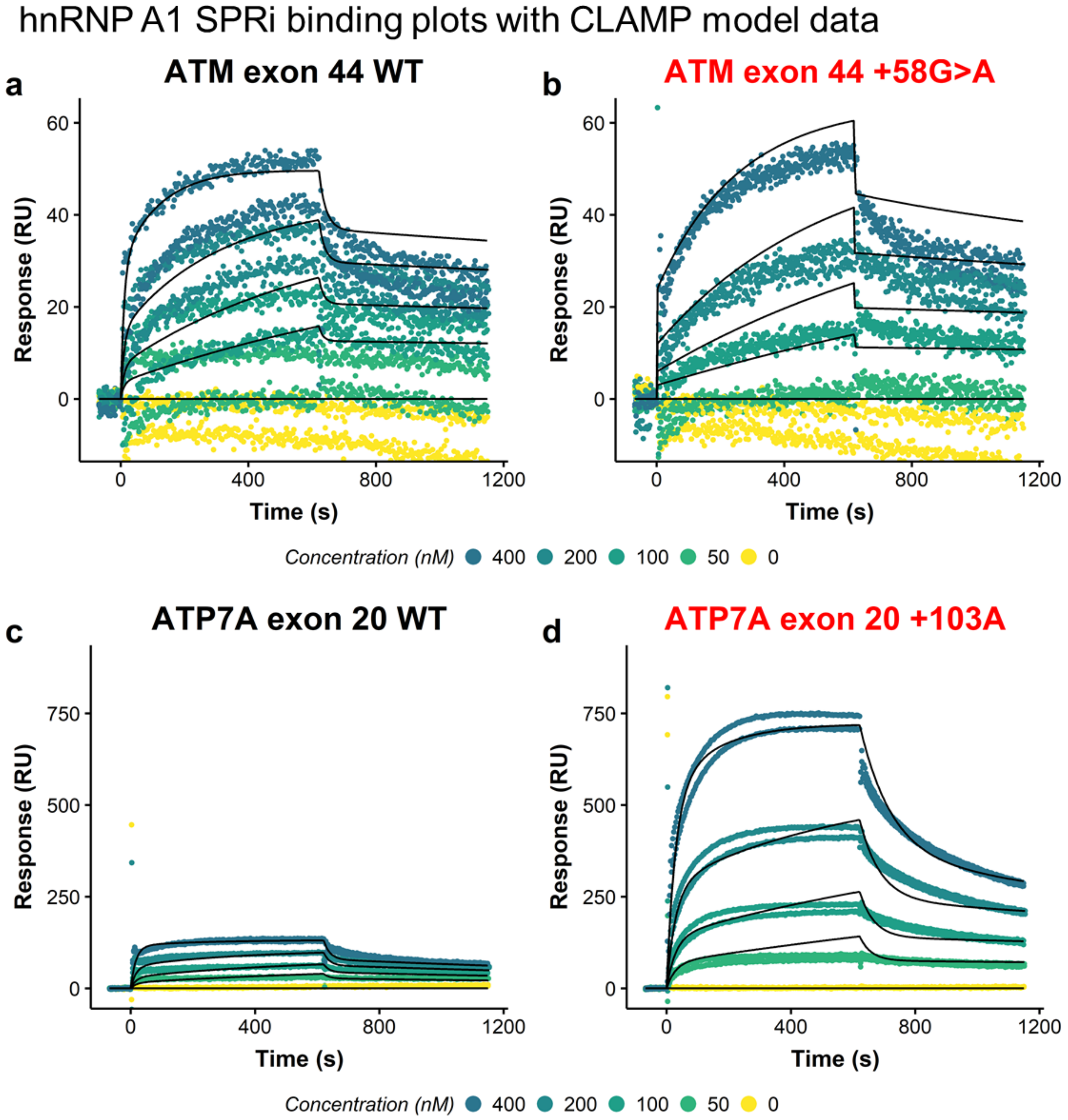

Figure S38 | Results of SPRi measurements of hnRNP A1 binding to a set of RNA wt and mutant oligos. Plots showing measured response (RU) versus time of the wt oligo (left) and mutant (right). The combined model fit across concentrations is indicated in black and concentrations are shown in decreasing order as gradually changing colors from blue through green to yellow. The fitted model's maximum simulated value is indicated above each plot.

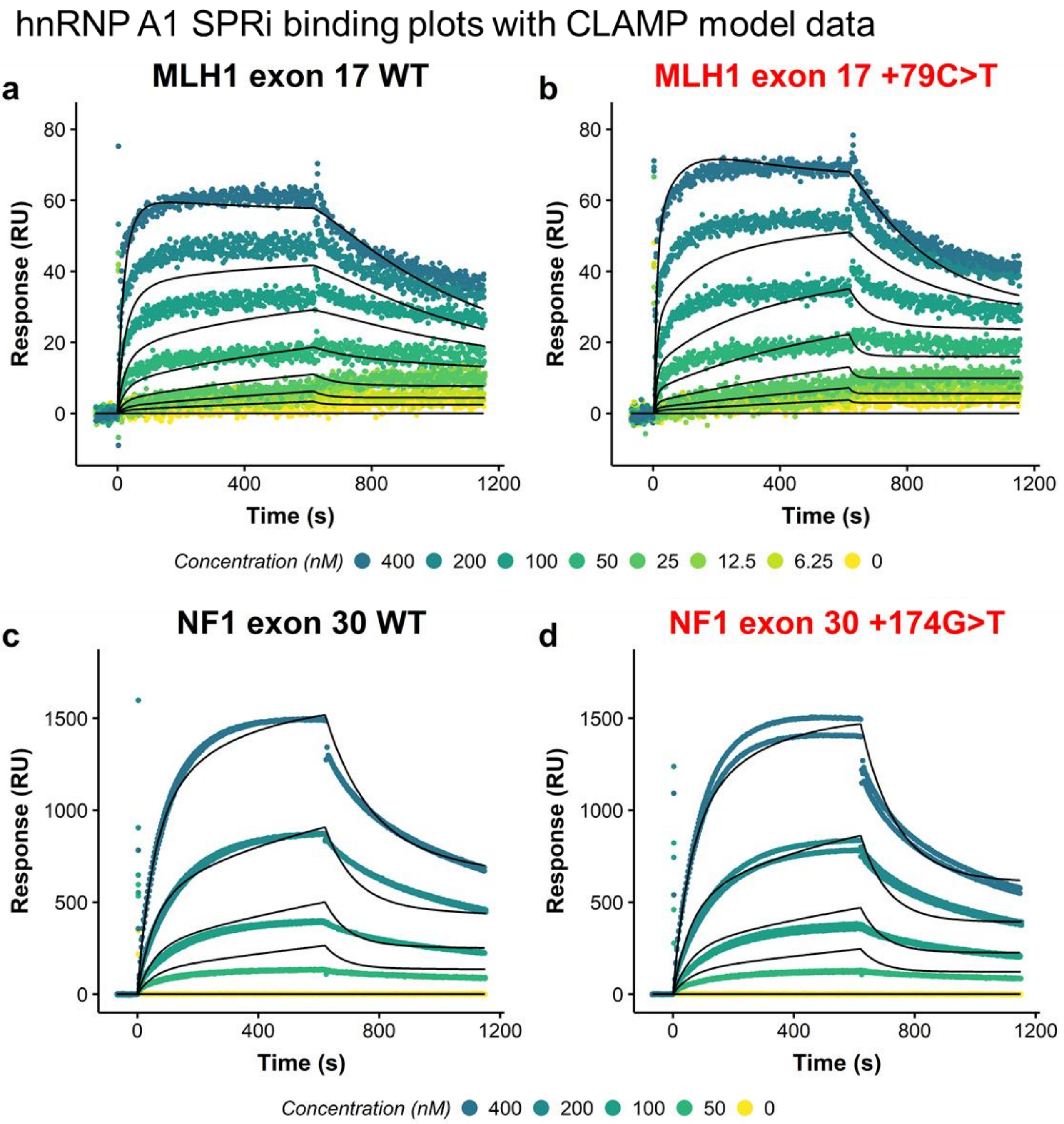

Figure S39 | Results of SPRi measurements of hnRNP A1 binding to a set of RNA wt and mutant oligos. Plots showing measured response (RU) versus time of the wt oligo (left) and mutant (right). The combined model fit across concentrations is indicated in black and concentrations are shown in decreasing order as gradually changing colors from blue through green to yellow. The fitted model's maximum simulated value is indicated above each plot.

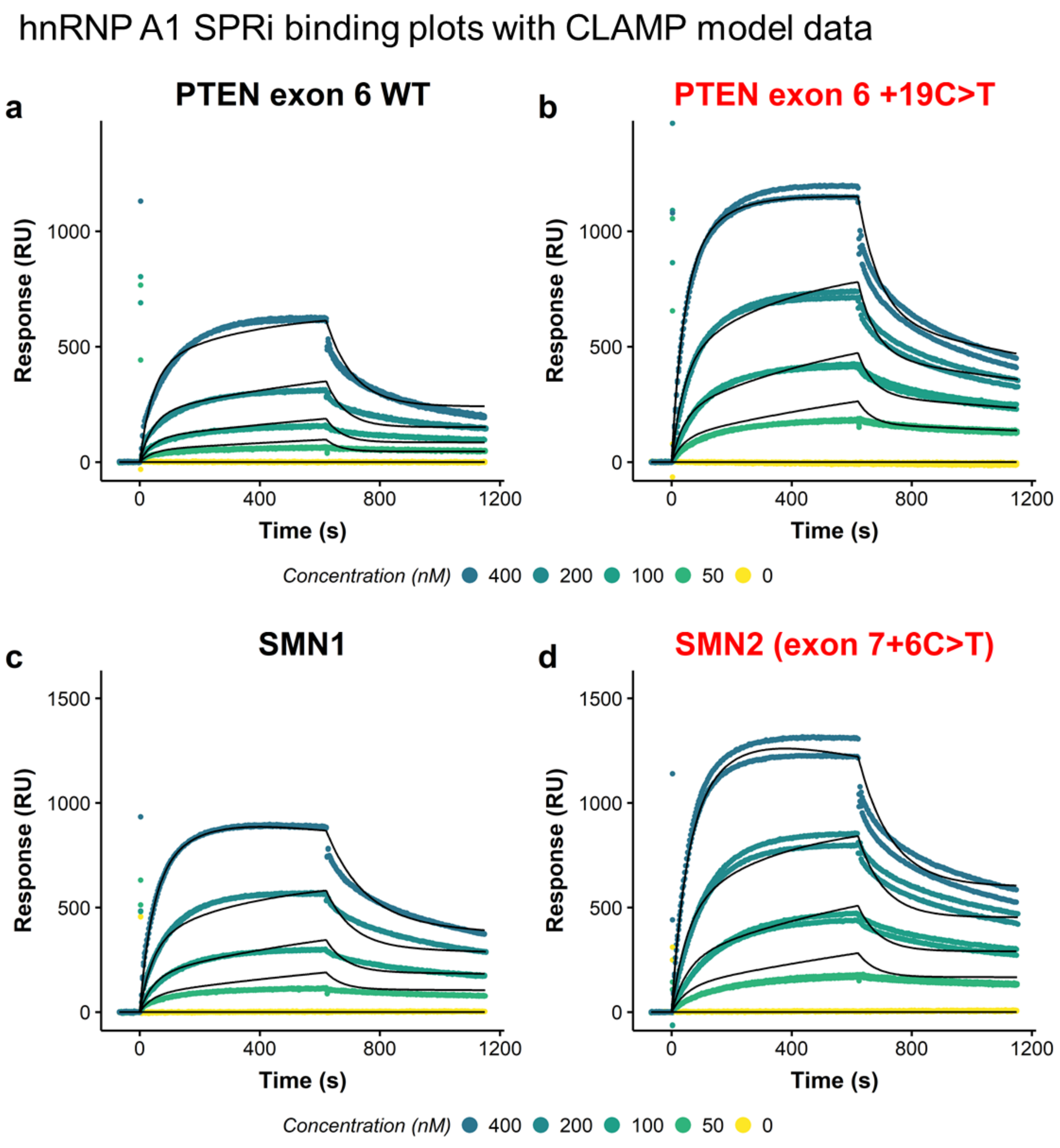

Figure S40 | Results of SPRi measurements of hnRNP A1 binding to a set of RNA wt and mutant oligos. Plots showing measured response (RU) versus time of the wt oligo (left) and mutant (right). The combined model fit across concentrations is indicated in black and concentrations are shown in decreasing order as gradually changing colors from blue through green to yellow. The fitted model’s maximum simulated value is indicated above each plot.

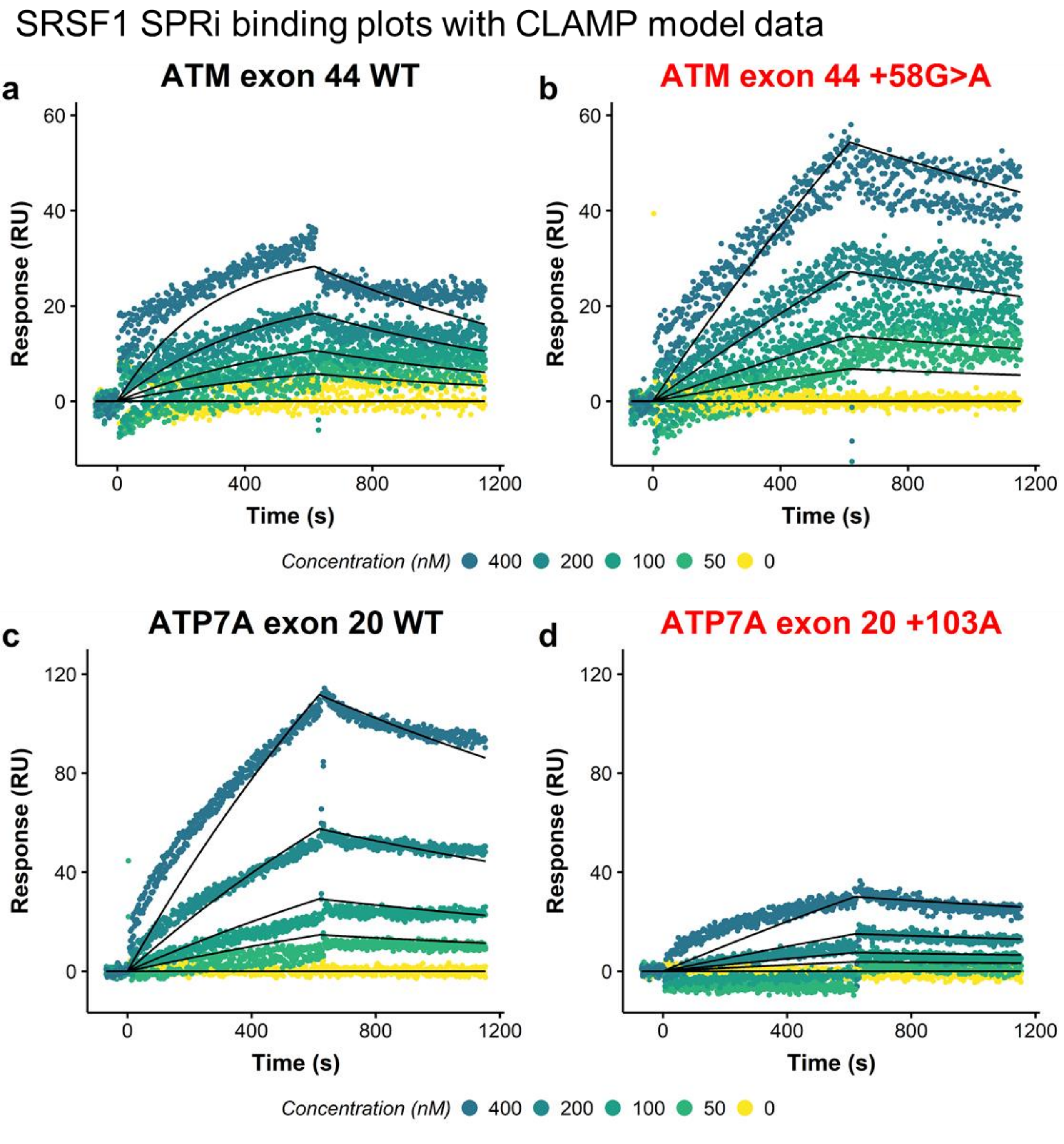

Figure S41 | Results of SPRi measurements of SRSF1 binding to a set of RNA wt and mutant oligos. Plots showing measured response (RU) versus time of the wt oligo (left) and mutant (right). The combined model fit across concentrations is indicated in black and concentrations are shown in decreasing order as gradually changing colors from blue through green to yellow. The fitted model’s maximum simulated value is indicated above each plot.

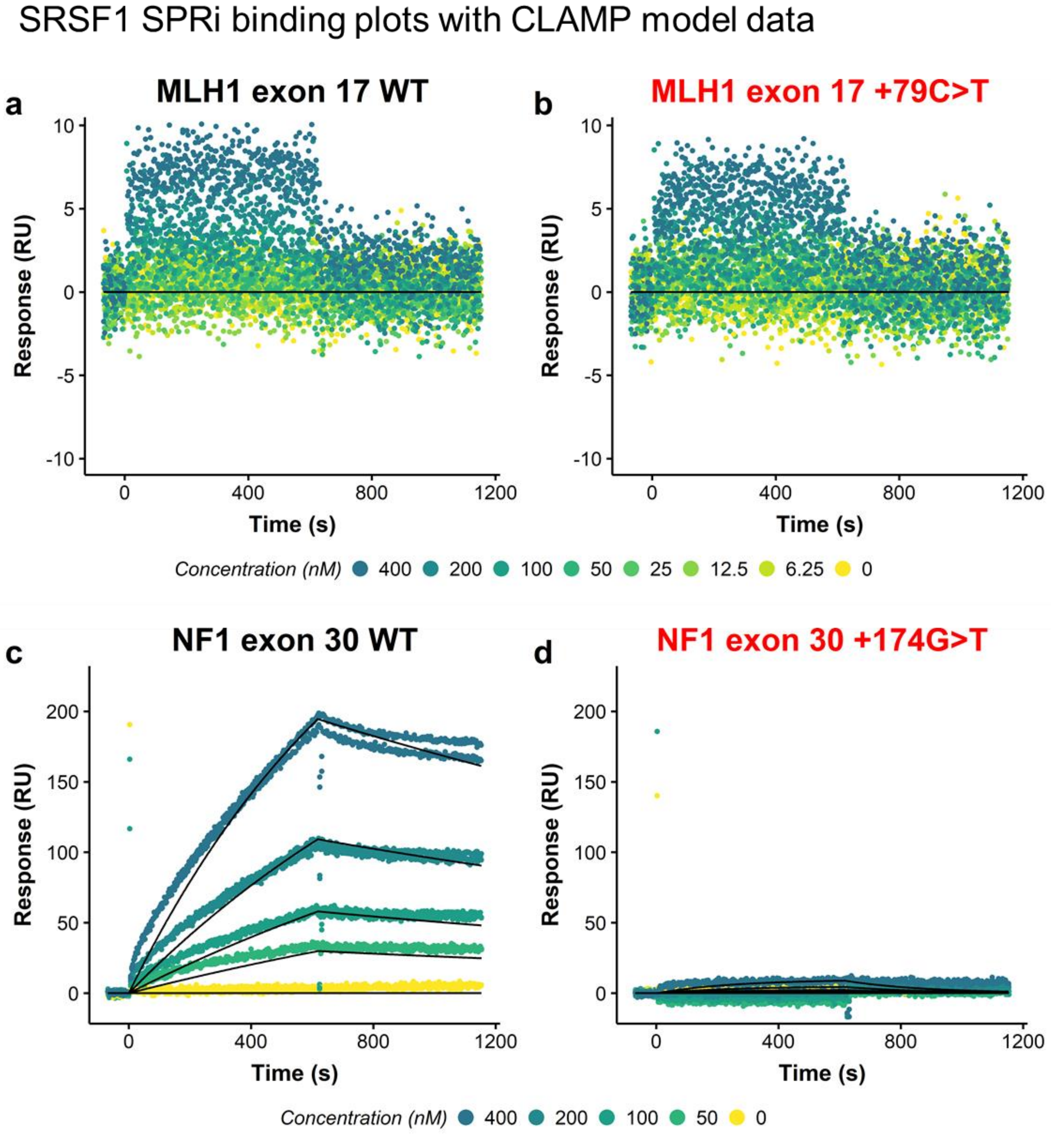

Figure S42 | Results of SPRi measurements of SRSF1 binding to a set of RNA wt and mutant oligos. Plots showing measured response (RU) versus time of the wt oligo (left) and mutant (right). The combined model fit across concentrations is indicated in black and concentrations are shown in decreasing order as gradually changing colors from blue through green to yellow. The fitted model's maximum simulated value is indicated above each plot.

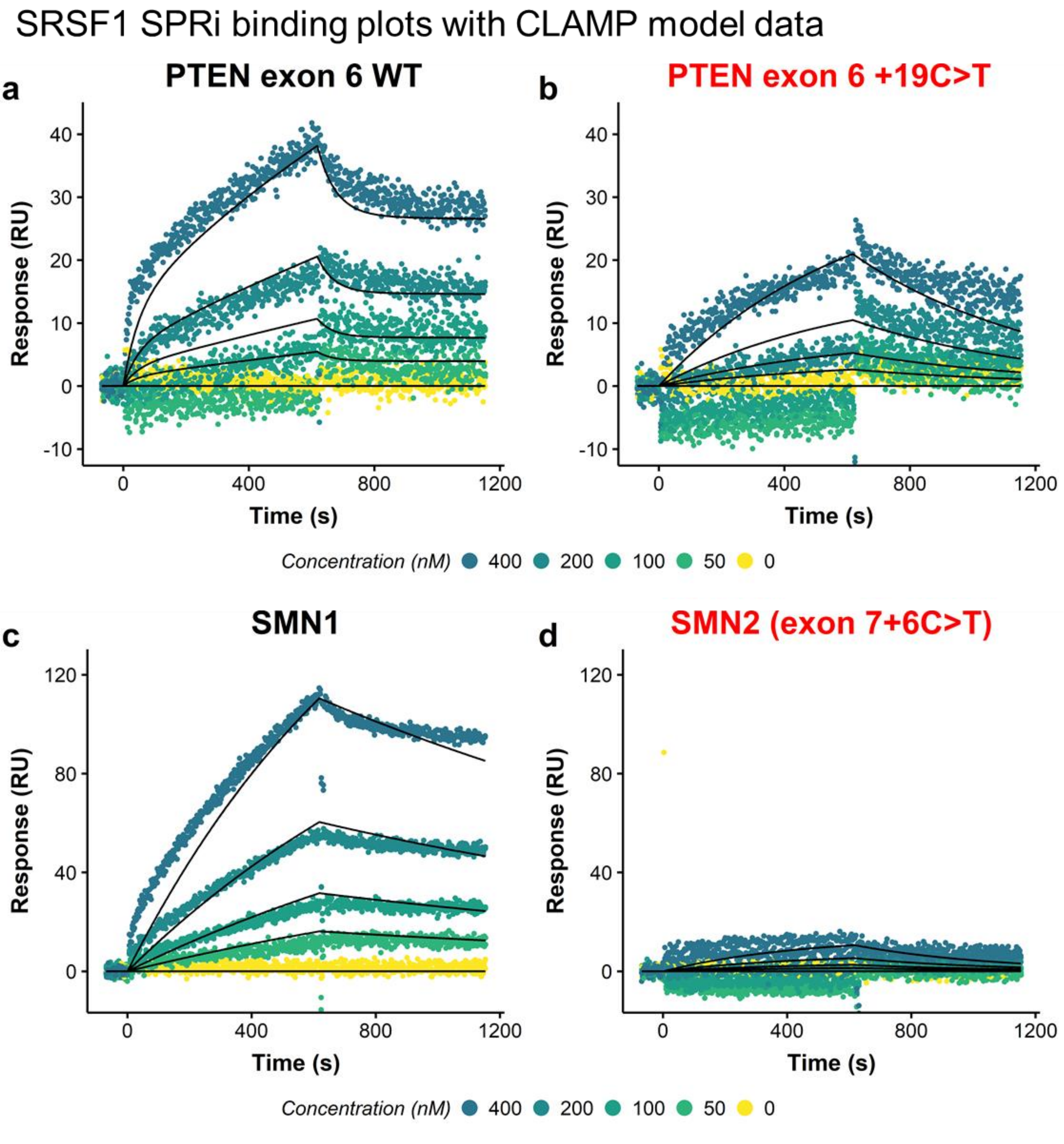

Figure S43 | Results of SPRi measurements of SRSF1 binding to a set of RNA wt and mutant oligos. Plots showing measured response (RU) versus time of the wt oligo (left) and mutant (right). The combined model fit across concentrations is indicated in black and concentrations are shown in decreasing order as gradually changing colors from blue through green to yellow. The fitted model's maximum simulated value is indicated above each plot.

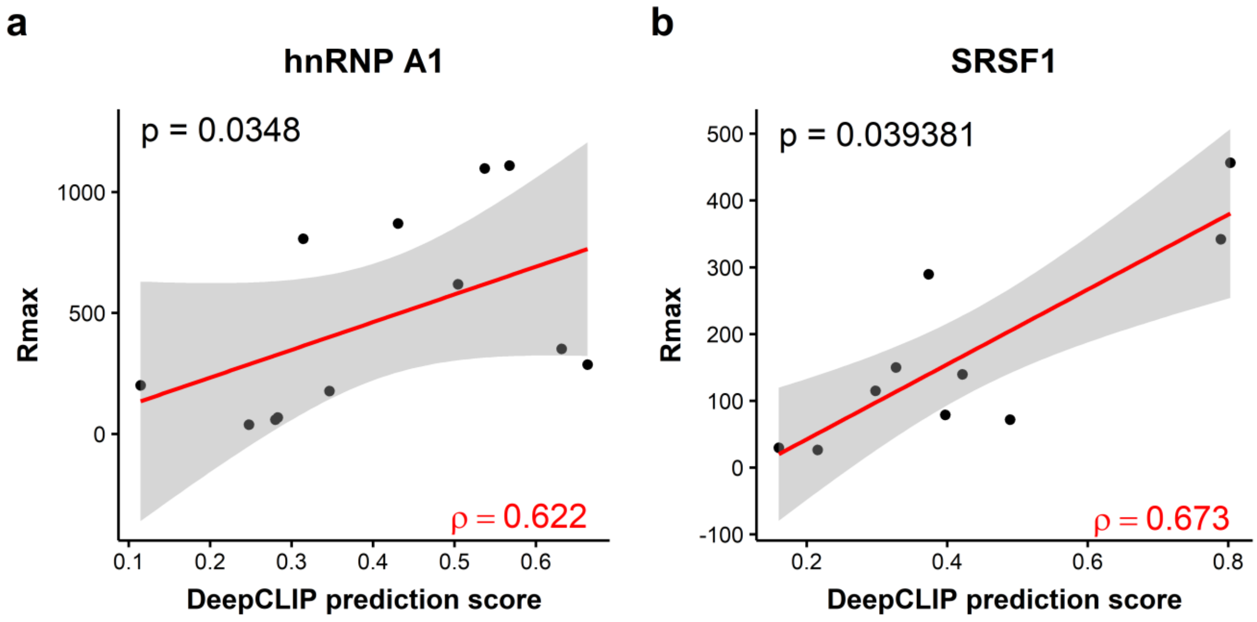

Figure S44 | Correlation between observed *in vitro* binding and DeepCLIP hnRNP A1 and SRSF1 model predictions. (a) Scatter plot of Rmax binding values from SPRi measurements of hnRNP A1 binding to oligos vs the predicted DeepCLIP hnRNP A1 scores. (b) Scatter plot of Rmax binding values from SPRi measurements of SRSF1 binding to oligos vs the predicted DeepCLIP SRSF1 scores. Spearman's rho is indicated in red in lower right corner and p-value in red in upper left corner for both plots in (a-b).

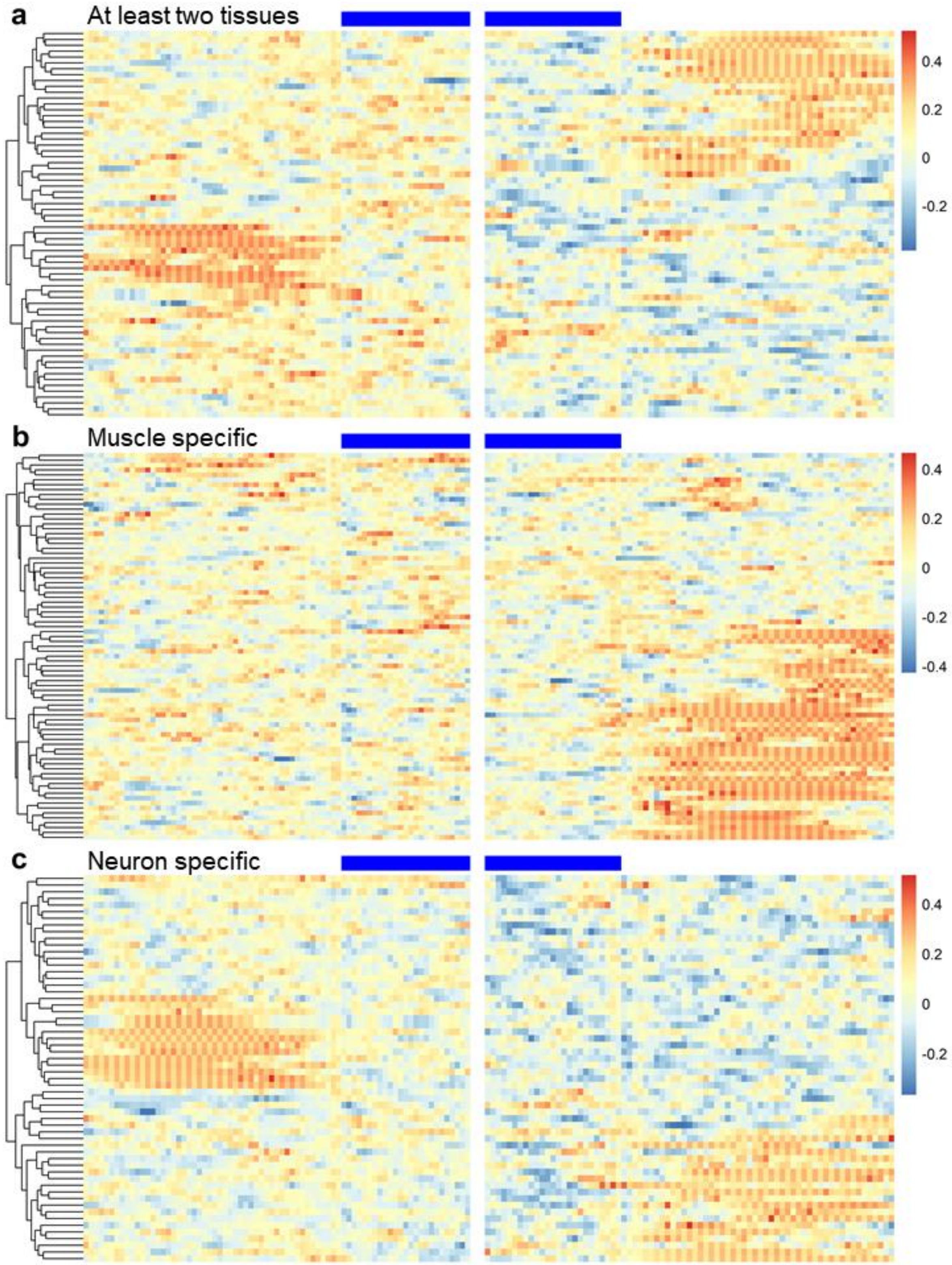

Figure S45 | DeepCLIP predicted binding at TDP-43 repressed pseudoexons. (a-c) Heatmap of TDP-43 DeepCLIP binding profiles at the 3'ss and 5'ss regions of TDP-43 repressed pseudoexons in mice. In all heatmaps the plots show 25 nt into the exon indicated by blue bars at the top, and the 50 first and last nt of the introns. The heatmap in (a) shows pseudoexons up-relugated upon TDP-43 depletion in at least two of the examined tissues (stem cells, neurons, and muscle cells), while the heatmap in (b) show muscle specific pseudoexons and the heatmap in (c) shows neuron specific pseudoexons.

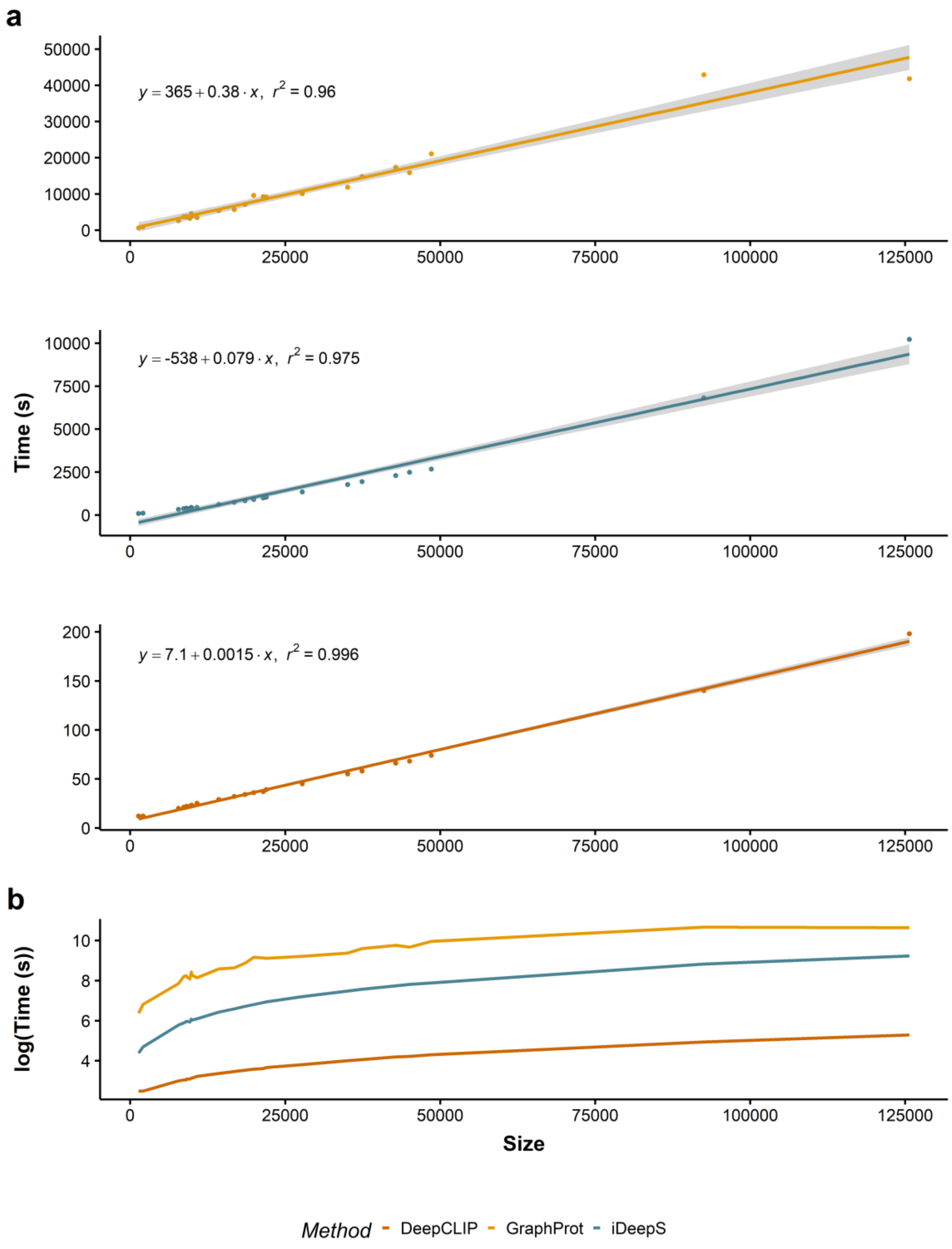

Figure S46 | Comparison of runtime lengths between GraphProt, iDeepS, and DeepCLIP. (a) Linear regression analysis of runtime lengths using the GraphProt benchmark dataset, each model timed on its own positive training data. GraphProt results are shown at the top, iDeepS in the middle and DeepCLIP at the bottom. The results of linear regression are indicated in each plot. (b) Log-linear plot of the data in (a) to compare the three methods on an equal scale. All methods were run on an Linux machine with an Intel(R) Xeon(R) CPU E5-2620 v4 @ 2.10GHz with 32G RAM and Tesla K40c GPU.

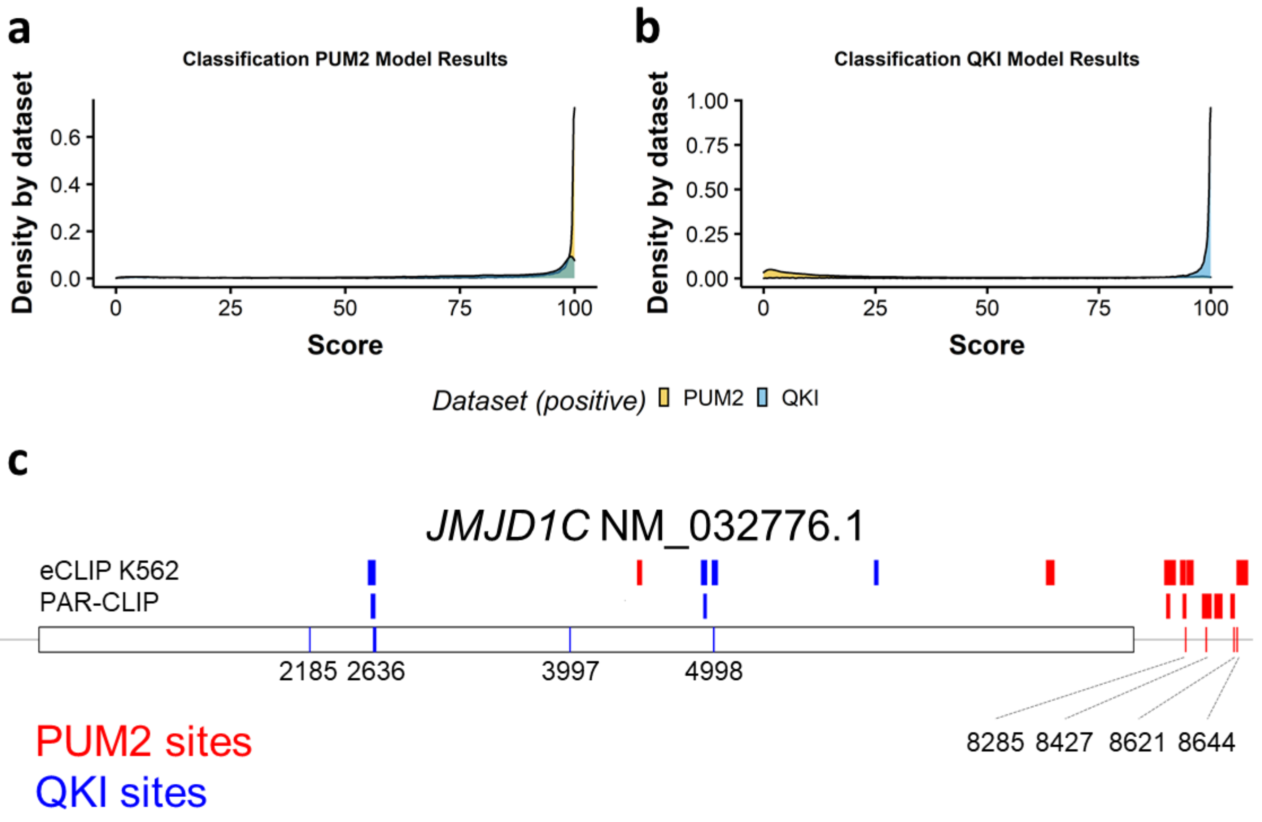

Figure S47 | Benchmark of PUM2 and QKI models. (a) The DeepCLIP PUM2 model was used to score the positive PUM2 dataset (yellow) and the positive QKI dataset (blue) and the distribution of scores was plotted as a density profile. (b) Same as (a), but using the DeepCLIP QKI model to score the two datasets. (c) The PUM2 model and QKI model were used in long-prediction mode to produce binding profiles across the length of the NM032776.1 transcript (8762 nt total). Predicted binding sites for each model were obtained by finding windows of at least 9nt length with a mean score of at least 0.3. PAR-CLIP and eCLOP sites are indicated above the transcript and DeepCLIP predicted sites within the transcript. The coding sequence of the transcript is indicated by a bar, and UTR regions by lines.
